# Antimutagenic and anticoagulant therapeutic effects of Ag/Ag_2_O nanoparticles from *Olea europaea* leaf extract: mitigating metribuzin-induced hepato-and nephrotoxicity

**DOI:** 10.3389/fphar.2024.1485525

**Published:** 2024-10-23

**Authors:** Manel Azzi, Ibtissam Laib, Abderrhmane Bouafia, Ifriqya Medila, Ali Tliba, Salah Eddine Laouini, Huda Alsaeedi, David Cornu, Mikhael Bechelany, Ahmed Barhoum

**Affiliations:** ^1^ Laboratory of Biology, Environment and Health, Faculty of Natural and Life Sciences, University of El Oued, El Oued, Algeria; ^2^ Department of Cellular and Molecular Biology, Faculty of Natural and Life Sciences, University of El Oued, El Oued, Algeria; ^3^ Department of Process Engineering and Petrochemical, Faculty of Technology, University of El Oued, El Oued, Algeria; ^4^ Laboratory of Biotechnology Biomaterials and Condensed Matter, Faculty of Technology, University of El Oued, El Oued, Algeria; ^5^ Lab. VTRS, Faculty of Technology, University of El Oued, El-Oued, Algeria; ^6^ Department of Chemistry, College of Science, King Saud University, Riyadh, Saudi Arabia; ^7^ Institut Européen des Membranes, IEM, UMR-5635, University Montpellier, ENSCM, CNRS, Place Eugene Bataillon, Montpellier, France; ^8^ Functional Materials Group, Gulf University for Science and Technology (GUST), Mubarak Al-Abdullah, Kuwait; ^9^ NanoStruc Research Group, Chemistry Department, Faculty of Science, Helwan University, Cairo, Egypt

**Keywords:** silver nanoparticles, silver oxide nanoparticles, plant extract, phytochemicals, oxidative stress, metribuzin-induced toxicity, hematological parameters, lipid profiles

## Abstract

**Background:**

Silver nanoparticles (Ag/Ag₂O NPs) have garnered attention for their potent antioxidant, antimicrobial, and anti-inflammatory properties, showing promise for therapeutic applications, particularly in mitigating chemical-induced toxicity.

**Objective:**

This study aimed to synthesize Ag/Ag₂O NPs using Olea europaea (olive) leaf extract as a green, eco-friendly reducing agent and evaluate their protective effects against metribuzin-induced toxicity in Wistar rats, focusing on oxidative stress, hematological parameters, and lipid profiles, with specific dose optimization.

**Methodology:**

Ag/Ag₂O NPs were synthesized using Olea europaea leaf extract, and their properties were confirmed via XRD, FTIR, SEM, EDS, and UV-visible spectroscopy. Wistar rats exposed to metribuzin (110 mg/kg/day) were treated with two doses of Ag/Ag₂O NPs (0.062 mg/kg and 0.125 mg/kg). Hematological and biochemical markers were assessed to evaluate the NPs’ protective effects.

**Results:**

Physicochemical characterization confirmed the successful formation of Ag/Ag₂O NPs loaded with phytochemicals, exhibiting crystallite sizes of 23 nm and 19 nm, a particle size of 25 nm, and significant peaks in XRD, FTIR, and UV-Vis spectra indicating the formation of Ag/Ag₂O. Metribuzin exposure led to significant hematological disruptions (elevated WBC, reduced RBC and hemoglobin) and worsened lipid profiles (increased cholesterol, LDL, and triglycerides). The lower NP dose (0.062 mg/kg) improved WBC, RBC, hemoglobin, and platelet counts, normalized lipid levels, and positively influenced biochemical markers such as serum creatinine and uric acid. In contrast, the higher NP dose (0.125 mg/kg) showed mixed results, with some improvements but an increase in triglycerides and continued elevation of ASAT and ALAT enzyme levels.

**Conclusion:**

Ag/Ag₂O NPs synthesized via green methods using olive leaf extract effectively mitigated metribuzin-induced toxicity, especially at lower doses, by improving oxidative stress markers and hematological and biochemical profiles. Dose optimization is crucial to maximize therapeutic benefits and minimize adverse effects, underscoring their potential in treating chemical-induced toxicity.

## 1 Introduction

Mutagenic agents present a substantial global health risk due to their ability to induce DNA mutations, which play a crucial role in the development of cancer. These mutations can lead to genomic instability, altered gene expression, and disruption of critical cellular pathways, ultimately contributing to carcinogenesis (Basu, 2018). The biochemical mechanisms through which mutagens exert their effects involve the formation of DNA adducts and the generation of reactive oxygen species (ROS), which cause oxidative damage to cellular macromolecules. This oxidative stress can lead to mutations in oncogenes and tumor suppressor genes, further driving the cancer process. Environmental pollutants and lifestyle factors are significant contributors to the prevalence of mutagenic agents. For instance, tobacco smoke is a major source of exposure to a complex mixture of over 7,000 chemicals, many of which are known carcinogens. Among these, benzene and formaldehyde are prominent due to their ability to form highly reactive metabolites that can covalently bind to DNA, leading to mutagenic lesions and genomic instability (Mavragani et al., 2017). The continuous exposure to these carcinogens is linked to various cancers, including lung, bladder, and throat cancers, and contributes to approximately 1.8 million new cancer cases annually (Pathak et al., 2022).

Thrombotic disorders, including conditions like deep vein thrombosis and pulmonary embolism, affect hundreds of thousands of individuals each year and are typically managed with anticoagulants like warfarin and heparin (Kaur et al., 2024). While effective, these treatments come with significant risks, such as bleeding complications, necessitating continuous monitoring (Hernández et al., 2013). Therefore, there is a critical demand for safer and more efficient therapeutic alternatives. Phytochemical-loaded nanoparticles (NPs) offer a promising solution in this context (Wasef et al., 2021). These advanced drug delivery systems enhance the stability, bioavailability, and targeted delivery of therapeutic agents, potentially reducing the adverse effects associated with conventional treatments (Osman et al., 2024). Incorporating medicinal plant extracts into NPs provides a powerful strategy to combat metribuzin-induced hepatotoxicity and nephrotoxicity (Ibtissam and Boutlilis, 2023). Phytochemicals like curcumin and resveratrol, known for their anticancer and antioxidant properties, show enhanced therapeutic efficacy when encapsulated in NPs, overcoming challenges like poor bioavailability (Khan et al., 2019). This approach not only amplifies their beneficial effects but also ensures targeted delivery to affected tissues, crucial for mitigating the toxic effects of substances like metribuzin.

Among the diverse medicinal plants used for green synthesis, *Olea europaea* (olive) is particularly esteemed due to its rich phytochemical profile and long-standing therapeutic history. Olive leaves, in particular, are abundant in bioactive compounds such as oleuropein, hydroxytyrosol, flavonoids, and phenolic acids, which contribute to their powerful antioxidant, antimicrobial, and anti-inflammatory properties (El and Karakaya, 2009; Magyari-Pavel et al., 2024). These compounds not only provide protection against oxidative stress but have also shown significant therapeutic potential in treating conditions such as diabetes, hypertension, inflammation, diarrhea, respiratory and urinary tract infections, gastrointestinal disorders, asthma, hemorrhoids, rheumatism, and they are used as a laxative, mouth cleanser, and vasodilator (Castejon et al., 2020; Reboredo-Rodriguez et al., 2018). However, some studies have reported side effects, including coughing, dizziness, stomach pain, and headaches. Additionally, individuals allergic to olive tree pollen may experience allergic reactions when using olive leaf extract. In green nanotechnology, the medicinal properties of *Olea europaea* have been harnessed, with its leaf extract being increasingly used as a natural reducing agent in the synthesis of NPs (Felimban et al., 2022; Hashemi et al., 2016). This method reduces the need for harmful chemicals and improves the biocompatibility of the NPs, making them ideal for biomedical applications.

Ag/Ag_2_O NPs particularly when synthesized through green methods using various plant extracts exhibit potent antioxidative, anti-inflammatory, and antimicrobial properties (Gangal et al., 2024). Ag/Ag_2_O NPs synthesized using Moringa oleifera leaf extract have been proven effective in reducing oxidative stress markers and restoring normal liver and kidney histology in cadmium-exposed rats (Mohammed and Hawar, 2022). Similarly, Ag/Ag_2_O NPs derived from Catharanthus roseus (Madagascar periwinkle) have demonstrated selective cytotoxicity against breast cancer cells (Muskan, 2022). The use of Azadirachta indica (Neem) has yielded Ag NPs with potent apoptotic effects against lung cancer cells (Roy et al., 2017). Another noteworthy study demonstrated the therapeutic potential of Ag NPs synthesized from Helianthemum lippii extract in mitigating cadmium-induced hepatotoxicity in Wistar rats (Laib et al., 2024b). Moreover, the green synthesis approach, exemplified by the use of Ocimum sanctum (Holy basil), Curcuma longa (Turmeric), and Zingiber officinale (Ginger), has further emphasized the versatility and potency of plant-mediated Ag NPs, contributing to their superior antibacterial, antioxidant, and anticancer properties (Venkatadri et al., 2020).

The aim of this study is to synthesis and evaluate *Olea europaea* (olive) leaf extract-based Ag/Ag₂O NPs as a safer, environmentally friendly therapeutic approach for mitigating toxicities, in line with green chemistry principles. For the methodology, *Olea europaea* leaf extract was employed as an eco-friendly reducing and capping agent to synthesize the Ag/Ag₂O NPs. This green synthesis approach leverages the bioactive compounds and antioxidants naturally present in the olive leaf extract to enhance the stability and bioavailability of the Ag/Ag₂O NPs. The research includes both *in vitro* assays to evaluate the NPs’ ability to prevent DNA mutations caused by mutagenic agents, and *in vivo* experiments to assess their impact on metabolic parameters and tissue health in Wistar rats exposed to metribuzin. Biochemical markers, oxidative stress levels, and histopathological changes in the liver and kidneys are analyzed to understand the therapeutic effects of the Ag/Ag₂O NPs on metabolic disturbances. The novelty and importance of this research lie in the integration of *Olea europaea* leaf extract’s traditional medicinal value with modern nanotechnology, creating a multifunctional therapeutic agent. By combining the bioactivity of olive leaf phytochemicals with the properties of Ag/Ag₂O NPs, this approach offers a potent solution for addressing metribuzin-induced toxicity and oxidative stress. This study not only advances the application of plant-based Ag/Ag₂O NPs in biomedicine but also emphasizes the relevance of green synthesis methods, reducing reliance on hazardous chemicals while improving therapeutic efficacy.

## 2 Experimental

### 2.1 Chemicals and reagents

In this study, high-purity chemicals and reagents were employed to ensure precise experimental conditions. These included Metribuzin (C₈H₁₄N₄OS, 98%), silver nitrate (AgNO₃, 99.9%), potassium dihydrogen phosphate (KH₂PO₄, 99.5%), and dibasic potassium phosphate (K₂HPO₄, 99.95%). Ethylenediaminetetraacetic acid (EDTA, 99.0%), 2-thiobarbituric acid (TBA, 97.0%), and salicylic acid (C₇H₆O₃, 95.5%) were utilized for oxidative stress assays, while 5,5′-dithiobis-2-nitrobenzoic acid (DTNB, 98.28%) was used for glutathione (GSH) measurements. Other key reagents included 4-nitro blue tetrazolium chloride (NBT, 99.9%), ethanolamine (C₂H₇NO, 99%), o-cresolphthalein (C₂₂H₁₈O₄, 95%), 8-hydroxyquinoline (C₉H₇NO, 99.99%), Tris buffer (pH 7.8), and hydrogen peroxide (H₂O₂, 99.9%). Reagents like sodium hydroxide (NaOH, 99%), picric acid (C₆H₃N₃O₇, 99.8%), dimethyl sulfoxide (DMSO, ≥99.9%), and chloroform (CHCl₃, 94%) were also used. All chemicals were sourced from reputable suppliers and were used without further purification. Fresh olive leaves (*Olea europaea L*. var. Chemlali) were collected in April 2022 from the Nakhla region, El-Oued province, Algeria (GPS coordinates: 33° 22′06″N, 6° 52′03″E). The leaves were authenticated by a botany specialist and stored under cool, dry conditions away from light at room temperature before use.

### 2.2 Phytochemical extract and green synthesis of Ag/Ag₂O NPs

The *Olea europaea* leaves were first washed with distilled water, then dried for 12 days in a shaded area at room temperature before being crushed into a fine powder. To prepare the extract, 10 g of the powdered olive leaves were mixed with 100 mL of distilled water in a 250 mL glass beaker. The mixture was stirred continuously at room temperature for 24 h. Following this, the extract was filtered using Whatman No. 42 filter paper and stored in a glass container at 4°C for future use, with a maximum storage time of 1 month under dark conditions. The synthesis of Ag/Ag₂O NPs was conducted using a green synthesis method (Laouini et al., 2021). In a 250 mL Erlenmeyer flask, 1 mL of *Olea europaea* leaf extract was mixed with 45 mL of 1 mM AgNO₃ solution and incubated at 75°C while shaking at 150 rpm for 2 hours. The progression of nanoparticle biosynthesis was visually monitored by the color change of the mixture from bright yellow to dark brown, indicating the reduction of silver ions to Ag/Ag₂O NPs. The reaction mixture was left to stand at room temperature for 24 h to ensure complete nanoparticle formation. Throughout the experiment, controls were maintained with AgNO₃ solution and aqueous leaf extract alone. The nanoparticle solution was centrifuged at 3,000 rpm for 10 min to remove unbound phytochemicals and other impurities. The collected Ag/Ag₂O NPs were washed three times with distilled water to ensure purity and then dried overnight at 50°C. The resulting NPs were stored in containers for further characterization (Bouafia et al., 2021a).

### 2.3 Characterization of Ag/Ag_2_O NPs

The Ag/Ag₂O NPs were characterized using several analytical techniques. UV-visible spectroscopy (Shimadzu UV-2450) was used to evaluate their optical properties across a wavelength range of 200–800 nm, providing insights into their absorbance and potential bandgap energies. Fourier Transform Infrared Spectroscopy (FTIR) was employed to identify the functional groups in the NPs using the Thermo Scientific Nicolet iS5 FTIR spectrometer. Spectra were collected in the range of 4,000–400 cm⁻^1^, employing a KBr pellet method. The crystallinity and grain size of the NPs were determined using powder X-ray diffraction (XRD) with a MiniFlex 600 Rigaku X-ray diffractometer. The diffraction data were collected using CuKα radiation (λ = 1.5406 Å) across a 2θ range of 25°–90°. The Scherrer equation was used to calculate the crystallite size based on the full-width at half-maximum (FWHM) of the highest intensity peak. Scanning electron microscopy (SEM, TESCAN VEGA3 model) with energy dispersive X-ray spectroscopy (EDX) was employed to assess the surface morphology and elemental composition of the NPs. SEM analysis was conducted at an accelerating voltage of 10 kV, while the EDX resolution was 6 nm.

### 2.4 Antimutagenic activity assay

The antimutagenic properties of *Olea europaea*-mediated Ag/Ag₂O NPs were assessed using the Ames test with *Salmonella typhimurium* TA98, which detects frame-shift mutations. NPs at concentrations of 50–250 μg/mL were mixed with a mutagen and bacterial culture, then plated. After incubation, revertant colonies were counted to measure mutagenicity inhibition. The percentage inhibition (PI) was calculated with PI (%) = [(A- B)/A] × 100, where A is the revertant count in the mutagen-only control and B is in the presence of NPs. NPs were classified as strong (PI ≥ 40%), moderate (PI = 25–40%), or weak (PI ≤ 25%) based on their effectiveness (Saygi and Usta, 2021).

### 2.5 Anticoagulant activity test

The anticoagulant activity of the synthesized Ag/Ag₂O NPs was examined following the Raja et al. (2015) protocol. Blood samples were drawn from a healthy volunteer and collected into vials labeled A, B, C, and D. Vial A served as the negative control (without anticoagulants), while vials B and C were treated with NPs at 0.5% (v/v) concentration in blood (150 ppm). Vial D contained EDTA and acted as the positive control. Clotting times were measured to assess the anticoagulant effects of the NPs compared to controls (Raja et al., 2015).

### 2.6 Animal procurement and housing

Thirty-five male Wistar albino rats, weighing 222.35 ± 2.91 g, were obtained from the Pasteur Institute’s animal facility in Algiers, Algeria. The animals were housed in the Department of Molecular and Cellular Biology at the University of El-Oued, Algeria, under standardized conditions: relative humidity of 64%, temperature of 19°C, and a 12-h light/dark cycle. The rats were given *ad libitum* access to standard rat chow and water and were allowed to acclimatize for 2 weeks before the experiment. All animal procedures were approved by the Institutional Animal Ethics Committee (IAEC) at the University of El-Oued and followed ethical guidelines.

### 2.7 Metribuzin toxicity experiment

The Wistar rats were randomly divided into four groups (n = 5 per group) and subjected to the following treatments over a 3-week period:

• Group I: Control group receiving standard diet and water.
• Group II: Metribuzin administered orally at 110 mg/kg body weight (1/20 LD_50_).
• Group III: Metribuzin at 110 mg/kg body weight plus Ag/Ag_2_O NPs administered intraperitoneally at a dose of 0.0625 mg/kg.
• Group IV: Metribuzin at 110 mg/kg body weight plus Ag/Ag_2_O NPs administered intraperitoneally at a dose of 0.125 mg/kg. Daily intraperitoneal injections of physiological saline (0.9% NaCl) were administered to all groups.

### 2.8 Sub-acute toxicity assay

Sub-acute toxicity of the Ag/Ag_2_O NPs was evaluated in three groups of Wistar rats (n = 5 per group). Two groups received intraperitoneal injections of NPs at doses of 5 mg/kg and 2.5 mg/kg, respectively, while the control group received physiological saline (0.9%). Observations of mortality and behavioral changes were made daily for 28 days post-injection, noting symptoms like ataxia, lethargy, or physical discomfort.

### 2.9 Hematological parameters analysis

Hematological parameters were measured using Coulter’s method and analyzed with a Medonic automatic hematological analyzer (Coulter Beckman -USA-). Standard blood parameters such as red blood cell count, hemoglobin levels, and white blood cell differentials were computed for each rat.

### 2.10 Biochemical parameters analysis

The biochemical profile of each rat, including serum glucose, urea, uric acid, creatinine, cholesterol, and triglyceride levels, was assessed using commercial kits (Spinreact, Spain). Additionally, liver enzymes—glutamate-pyruvate transaminase (GPT) and glutamate-oxaloacetate transaminase (GOT)—were measured to evaluate liver function.

### 2.11 Oxidative stress parameters

Malondialdehyde (MDA) Levels were measured by combining 200 µL of the sample with 800 µL of TBA reagent in a glass test tube. The mixture was incubated in a water bath at 100°C for 15 min and then cooled in an ice bath for 30 min. After centrifugation at 3,000 rpm for 5 min, the absorbance of the supernatant was measured at 532 nm using a spectrophotometer. The concentration of thiobarbituric acid reactive substances (TBARS) was calculated and expressed as nmol/mg of protein (Reddy et al., 2017). Reduced Glutathione (GSH) levels were assessed using the Ellman reagent (2-nitro-5-thiobenzoic acid, DTNB). In this method, 200 µL of a 0.25% salicylic acid solution was mixed with 800 µL of homogenate samples, and the mixture was centrifuged at 1,000 rpm for 5 min. To 25 µL of DTNB (0.01 mol/L), 500 µL of the supernatant was added, along with 1,000 µL of tris buffer (0.4 mol/L tris, 0.02 mol/L NaCl, pH 8.9). After 5 min of incubation, the absorbance was measured at 412 nm. The GSH concentration was calculated using the provided equation and expressed in nanomoles per milligram of protein (nmol/mg of protein) (Nasr, 2014).

### 2.12 Histopathological examination

After euthanizing the rats, liver and kidney sections were collected and preserved in 10% formaldehyde until preparation for examination. The samples were washed with xylene, embedded in paraffin, and dehydrated through a graded series of ethanol (60%, 70%, 80%, and 100%). Using a Thermo Scientific (Micron HM 325) Histoline Rotary Microtome, paraffin blocks were sectioned into 4–6 µm slices, which were then stained with hematoxylin and eosin to enhance cellular details. Histopathological inspection was performed using a light microscope to evaluate tissue morphology.

### 2.13 Statistical analysis

Data are expressed as mean ± standard error (SE). Statistical analysis was performed using SPSS version 22.0, with significance assessed via One-Way ANOVA followed by Duncan’s multiple range test. Statistical significance was set at *p* ≤ 0.05.

## 3 Results and DISCUSSION

### 3.1 Crystal structure and composition of Ag/Ag_2_O NPs

**FIGURE 1:**
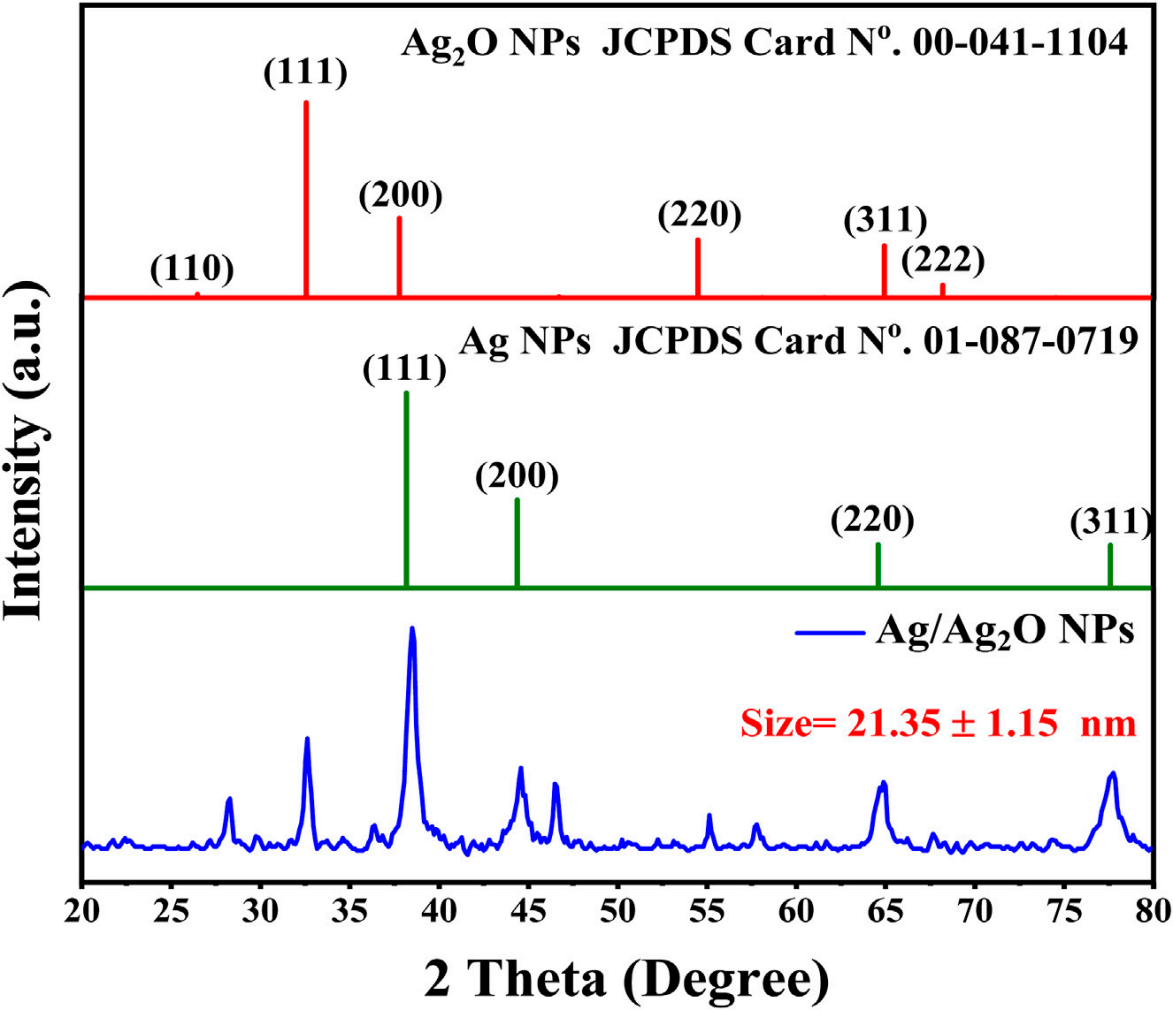
XRD Analysis of Ag/Ag₂O NPs synthesized from *Olea europaea* Leaf Extract.

Crystal structure and composition of the Ag/Ag_2_O nano-powder were extensively analyzed using X-ray diffraction (XRD), as illustrated in Figure 1. The XRD pattern reveals distinct peaks corresponding to both silver (Ag) and silver oxide (Ag₂O). Specifically, diffraction peaks at 2θ angles of 26.48°, 32.46°, 37.8°, 54.35°, 64.85°, and 68.15° correspond to the (110), (111), (200), (220), (311), and (222) planes of cubic Ag₂O, according to the Joint Committee on Powder Diffraction Standards (JCPDS Card No. 00-041–1,104) (Markgraf and Reader, 1985). Concurrently, peaks at 2θ values of 38.15°, 44.45°, 64.5°, and 77.65° match the (111), (200), (220), and (311) planes of face-centered cubic (fcc) metallic Ag (JCPDS Card No. 01-087–0,719) (Bouafia and Laouini, 2020). These results confirm the successful synthesis of Ag/Ag₂O NPs, distinctly identifying the silver and silver oxide phases. The well-defined peaks indicate high crystallinity, which is essential for their application in catalysis and electronic devices (Bouafia et al., 2021a). The average crystallite sizes of the NPs were determined using the Debye–Scherrer equation, revealing sizes of approximately 23.36 nm for Ag/Ag_2_O NPs (from peaks at 2θ values of 26.48°, 32.46°, and 37.8°) and 19.34 nm for Ag/Ag_2_O NPs(from peaks at 2θ values of 38.15° and 44.45°) (Bouafia and Laouini, 2021).

The leaf extract plays a key role in determining the crystal structure and size of Ag/Ag₂O NPs. It acts as a reducing agent, converting silver ions into metallic silver and facilitating silver oxide formation, leading to distinct Ag and Ag₂O phases. The extract also stabilizes the NPs, influencing their growth and ensuring the coexistence of both phases. Additionally, factors like pH and oxygen levels interact with the extract to shape the final structure, highlighting its importance in nanoparticle synthesis (Bouafia et al., 2021a).

Oumaima et *al*., (2019) conducted an HPLC-UV analysis of olive leaf extracts, revealing a rich phenolic profile that significantly contributes to the green synthesis of Ag/Ag₂O NPs. Among the identified compounds, oleuropein dominated, particularly in the ethanol-distilled water extract (80.67 ± 0.47 mg/g), where it plays a pivotal role in reducing silver ions to Ag/Ag₂O NPs and stabilizing them through its robust antioxidant properties. Gallic acid (2.44 ± 0.00 mg/g) and hydroxytyrosol (0.34 ± 0.00 mg/g) also contribute to the reduction process, enhancing the NPs’ stability and uniform morphology. In addition, rutin (1.15 ± 0.21 mg/g) and quercetin (0.37 ± 0.04 mg/g) assist in modifying the chemical environment during nanoparticle synthesis, ensuring optimal capping and preventing aggregation. The collective action of these phenolics not only facilitates the efficient formation of Ag/Ag₂O NPs but also imbues them with potent medicinal properties, making them highly effective in therapeutic applications. This comprehensive phenolic composition, especially in ethanol-distilled water extracts, underscores the potential of olive leaf-derived Ag/Ag₂O as a powerful, eco-friendly alternative in nanomedicine (Ghomari et al., 2019).

### 3.2 FTIR spectroscopy analysis

**FIGURE 2:**
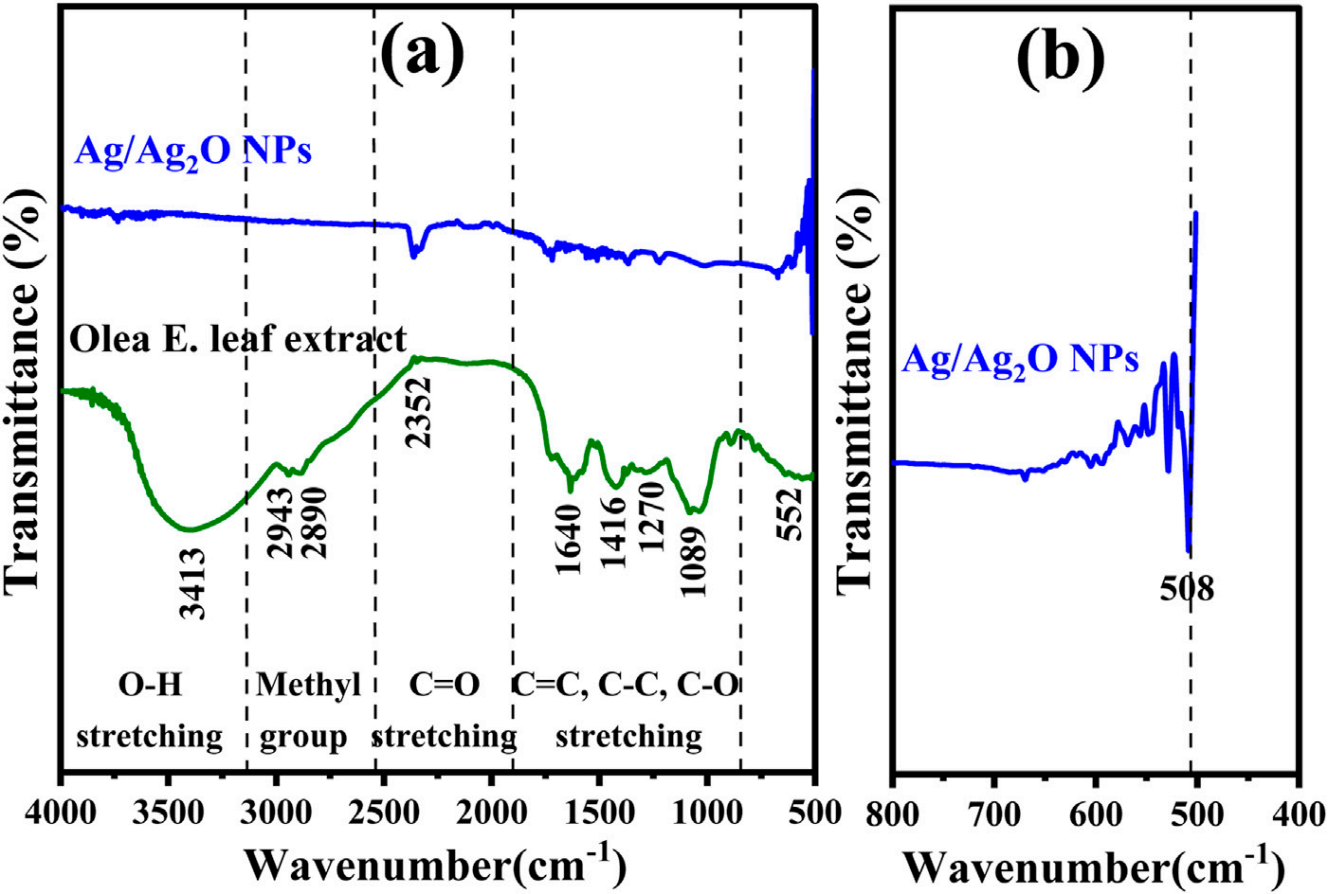
FT-IR Analysis of: **(A)** Ag/Ag_2_O NPs synthesized from *Olea europaea* Leaf Extract and *Olea europaea* leaf extract **(B)** Magnified spectrum (400–800 cm−^1^) of Ag/Ag_2_O NPs.

FTIR spectroscopy was employed to analyze the *Olea europaea* leaf extract and the synthesized Ag/Ag₂O NPs, aiming to identify the biomolecules responsible for the nanoparticle formation and verify their composition. The FTIR spectrum of the leaf extract, shown in Figure 2A, reveals several significant peaks: 3,413, 2,943, 2,890, 2,352, 1,640, 1,416, 1,270, 1,089, and 552 cm⁻^1^. The broad peak at 3,413 cm⁻^1^ is attributed to O-H stretching vibrations, indicative of hydroxyl groups commonly found in alcohols and phenolic compounds (Terea et al., 2023). These groups are crucial for their reducing and capping abilities during nanoparticle synthesis (Bouafia et al., 2021b). Peaks at 2,943 and 2,890 cm⁻^1^ correspond to C-H stretching vibrations of alkyl groups, reflecting the presence of lipid-like compounds in the extract (Laouini et al., 2021). The peak at 2,352 cm⁻^1^ represents the symmetric C=O stretching of CO₂, likely from atmospheric CO_2_ (Zidane et al., 2022). The peak at 1,640 cm⁻^1^ is associated with C=C stretching vibrations, suggesting the presence of flavonoids or aromatic compounds, which play a role in the reduction and stabilization of NPs (Ibtissam and Boutlilis, 2023). Additionally, the peak at 1,416 cm⁻^1^ is linked to C-C stretching in aromatic rings, indicating the presence of phenolic acids that may contribute to the reduction of metal ions (Chihi et al., 2023). The peak at 1,270 cm⁻^1^ corresponds to C-O stretching in carboxylic acids, which are essential for complex formation with metal ions, facilitating the reduction process (Allag et al., 2024). Peaks at 1,089 cm⁻^1^ and 552 cm⁻^1^ represent C-O-C stretching and out-of-plane bending vibrations, respectively, suggesting the presence of polysaccharides or phenolic compounds that stabilize the NPs (Kordy et al., 2022).

FTIR analysis confirms the successful synthesis of Ag/Ag₂O NPs Figure 2B, identifying key peaks at 618 cm⁻^1^ and 424 cm⁻^1^ that indicate the presence of silver oxide (Kordy et al., 2022). The analysis also highlights the role of functional groups in the *Olea europaea* extract, such as carbonyl, carboxyl, amide, and phenolic groups, which aid in reducing silver ions, stabilizing the Ag₂O NPs, and preventing their aggregation (Dobrucka et al., 2019).

### 3.3 Morphological investigation

**FIGURE 3:**
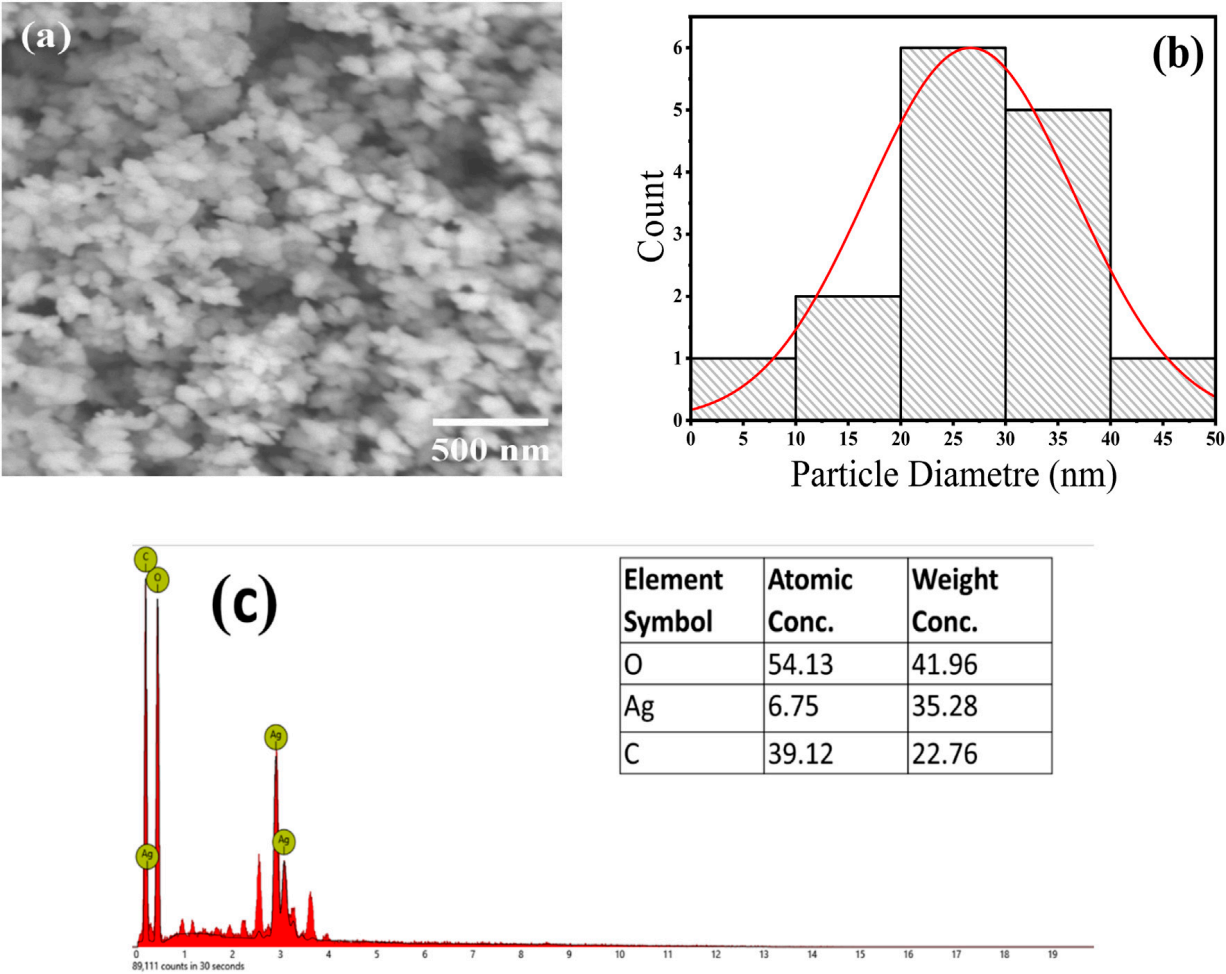
SEM analysis of the Ag/Ag₂O NPs Synthesized from *Olea europaea* Leaf Extract: **(A)** SEM images, **(B)** particle size diameter, **(C)** EDX elemental analysis.

SEM analysis of the Ag/Ag₂O NPs shows that they mainly have spherical to oval shapes, reflecting high morphological uniformity, as depicted in Figure 3A. This uniformity is attributed to the bioactive compounds in the *Olea europaea* leaf extract, which act as reducing and capping agents. These compounds control the size and shape of the NPs, leading to a consistent particle size of around 25 nm shown in Figure 3B. The narrow size distribution confirms the effectiveness of the biosynthesis process and the significant influence of the leaf extract’s phytochemicals on nanoparticle formation.

SEM-EDS analysis confirmed the formation and composition of Ag/Ag₂O NPs by showing peaks for silver (Ag), oxygen (O), and carbon (C), with atomic percentages of 6.75% for Ag, 54.13% for O, and 39.12% for C. The high oxygen content indicates significant oxidation, confirming the presence of Ag₂O phase. The carbon peak suggests that phytochemicals from the leaf extract are present on the nanoparticle surfaces, aiding in their stabilization and supporting the effectiveness of the green synthesis method (Figure 3C).

### 3.4 Bandgap and optical characteristics

**FIGURE 4:**
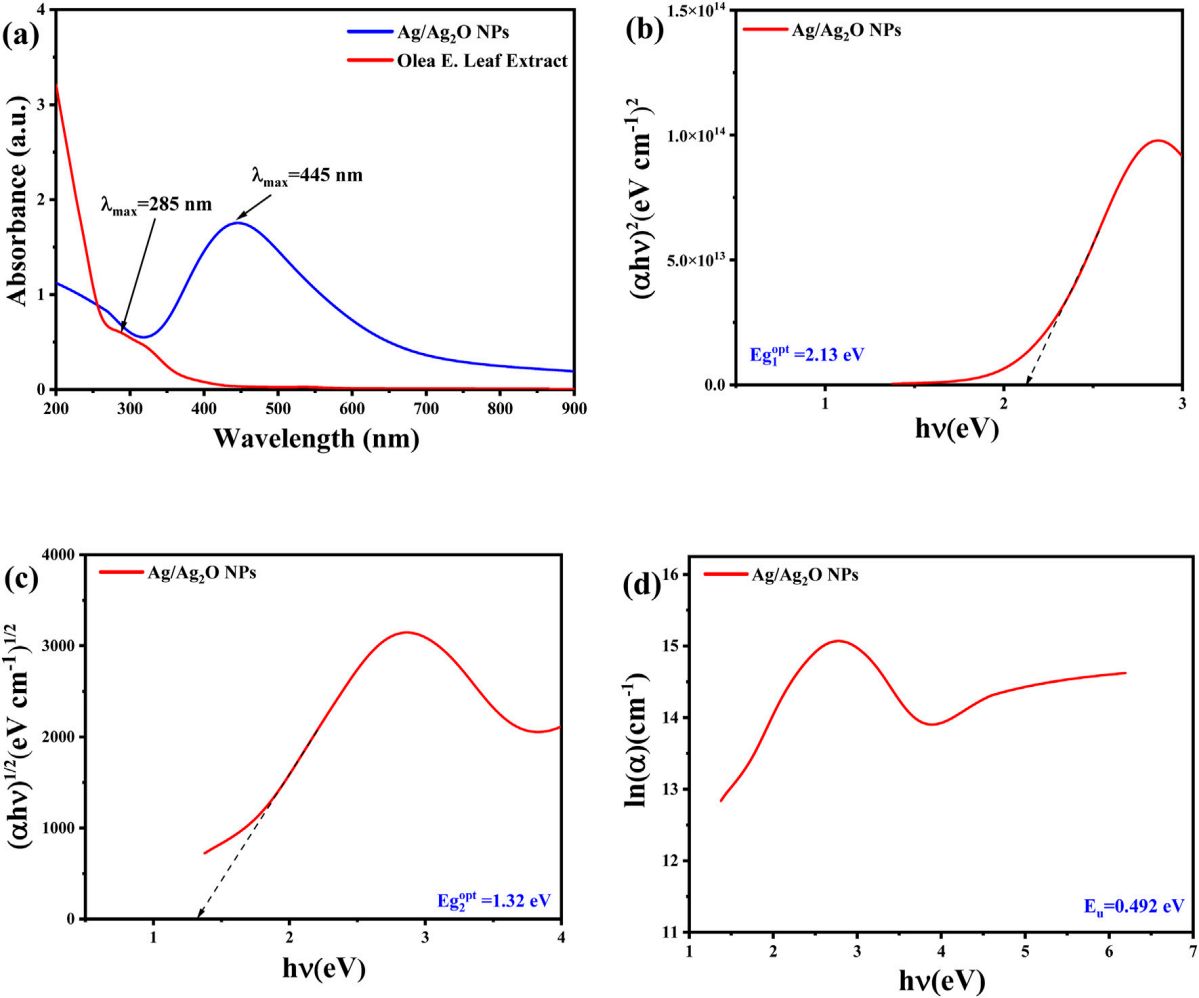
Optical properties of Ag/Ag₂O NPs synthesized from *Olea europaea* Leaf Extract: **(A)** UV-vis spectra **(B)** direct bandgap energy **(C)** indirect bandgap energy **(D)** the Urbach energy (Eu).

The UV-Vis absorption spectrum, shown in Figure 4A, reveals distinct optical features of both the *Olea europaea* leaf extract and the synthesized Ag/Ag₂O NPs. The plant extract displays a notable absorption peak at 285 nm, associated with phenolic compounds (Karan et al., 2024). In contrast, the synthesized Ag/Ag₂O NPs exhibit a prominent absorption peak at 445 nm, which is indicative of the unique electronic interactions involving Ag⁺ ions and nitrate ions (NO₃⁻) from the AgNO₃ precursor. This peak is crucial for confirming the presence of Ag/Ag₂O rather than pure silver, as the latter would typically show a peak around 400 nm (Bouafia et al., 2023). The shift to 445 nm supports the hypothesis that the bioactive components of the plant extract, particularly polyphenols with hydroxyl and ketone groups, facilitate the reduction of Ag⁺ to form Ag/Ag₂O NPs (Daoudi et al., 2022).

The energy band gap (E.g.,) values of the synthesized Ag/Ag₂O NPs were calculated using Tauc’s method, revealing direct and indirect band gaps of approximately 2.13 eV and 1.32 eV, respectively, as shown in Figures 4B,C. These band gap values are characteristic of Ag₂O and differ from those expected for pure silver, which typically has a higher band gap (Daoudi et al., 2022). The distinct band gap values and absorption peaks affirm the successful formation of Ag/Ag₂O NPs rather than pure Ag. Additionally, the Urbach energy (Eu), reflecting structural disorder, was determined from the absorption data, with a smaller Urbach energy indicating a more ordered structure (Figure 4D). This analysis further supports the formation of Ag/Ag₂O NPs by demonstrating optical properties consistent with the expected electronic structure of the composite material.

### 3.5 Antimutagenic activity

**FIGURE 5:**
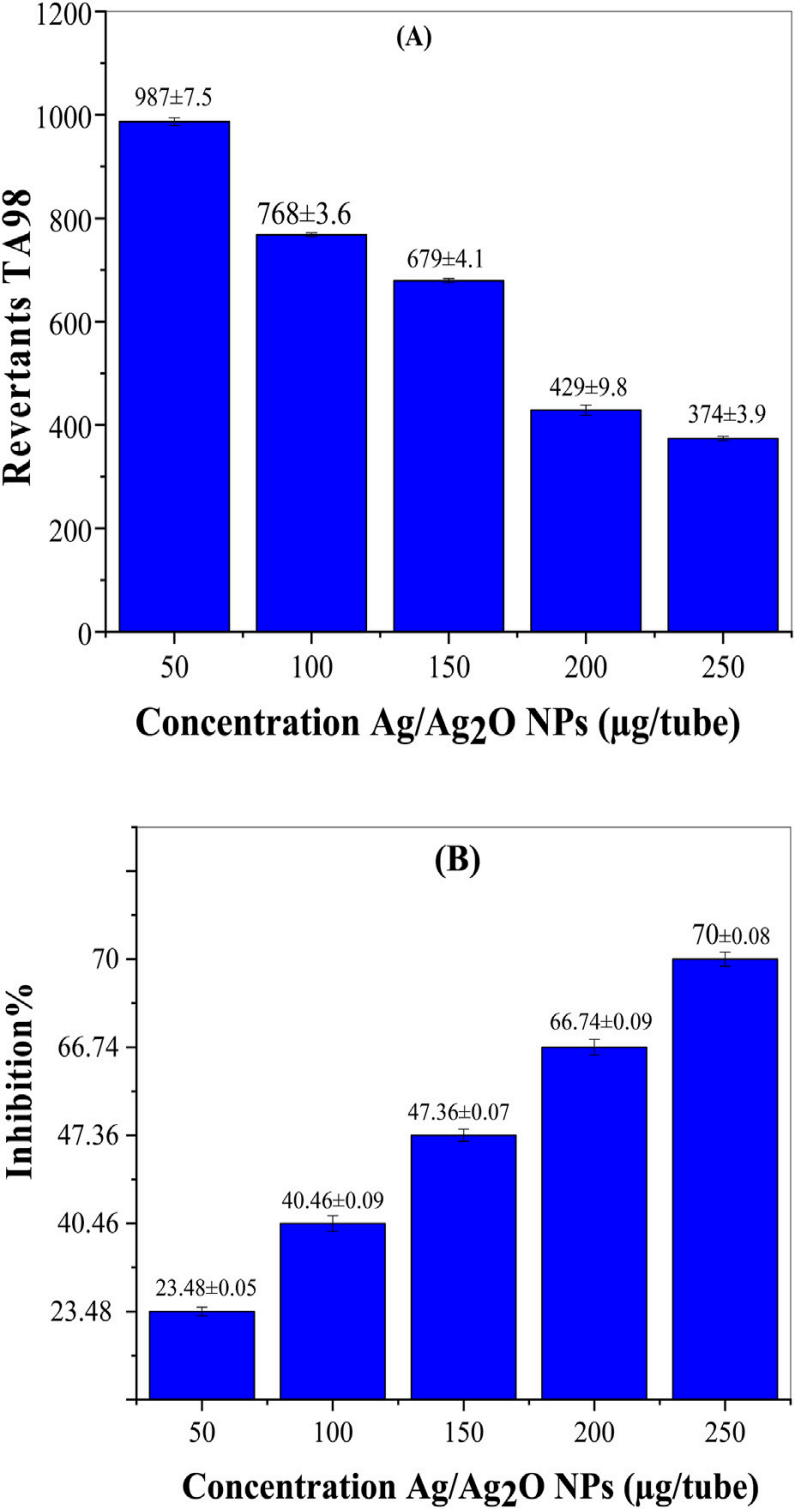
Antimutagenic activity of the Ag/Ag₂O NPs biosynthesized by aqueous extract of *O. europaea* leaves against 1-NP in *S. typhimurium* TA98. **(A)** Revertants TA98, **(B)** Inhibition %.

Figure 5 presents the antimutagenic effects of Ag/Ag₂O NPs synthesized from *Olea europaea* leaf extract. The positive control, 1-NP at 200 ng/tube, shows a high revertant count of 1,290 ± 9.8, indicating strong mutagenic activity. In contrast, Ag/Ag₂O NPs reduce revertant counts at various concentrations. At 50 µg/tube, there is a 23.48% reduction in mutagenicity, increasing to 70% at 250 µg/tube. This dose-dependent effect demonstrates that higher concentrations of Ag/Ag₂O NPs provide greater protection against mutations. The results align with literature suggesting that NPs can enhance biological activity due to their high surface area and reactivity, leading to more effective neutralization of mutagenic agents at higher doses (de Cássia Proença-Assunção et al., 2021).

The ability of Ag/Ag₂O NPs to enhance DNA repair mechanisms is crucial in restoring damaged DNA and preventing mutations (Heshmati et al., 2015). Maintaining genome integrity is fundamental to cellular function, and the potential of these NPs to influence DNA repair pathways opens new avenues for therapeutic applications (Khallef et al., 2019). Ag/Ag₂O NPs may activate multiple DNA repair pathways, enabling the restoration of damaged DNA strands and reducing the risk of mutations that could lead to cancer or other genetic disorders (Sarac et al., 2018). Future research should delve deeper into these mechanistic pathways to gain a better understanding of the antimutagenic and anticoagulant effects of these NPs. Clarifying these interactions will not only elucidate how Ag/Ag₂O NPs exert their protective effects but also guide the development of targeted therapeutic strategies. Further studies are necessary to identify specific molecular targets, signaling pathways, and the long-term impact of these NPs on cellular health. A comprehensive understanding of these factors is crucial for maximizing the safety and efficacy of Ag/Ag₂O NPs in clinical applications.

### 3.6 Anticoagulant activity of Ag/Ag_2_O NPs

**FIGURE 6:**
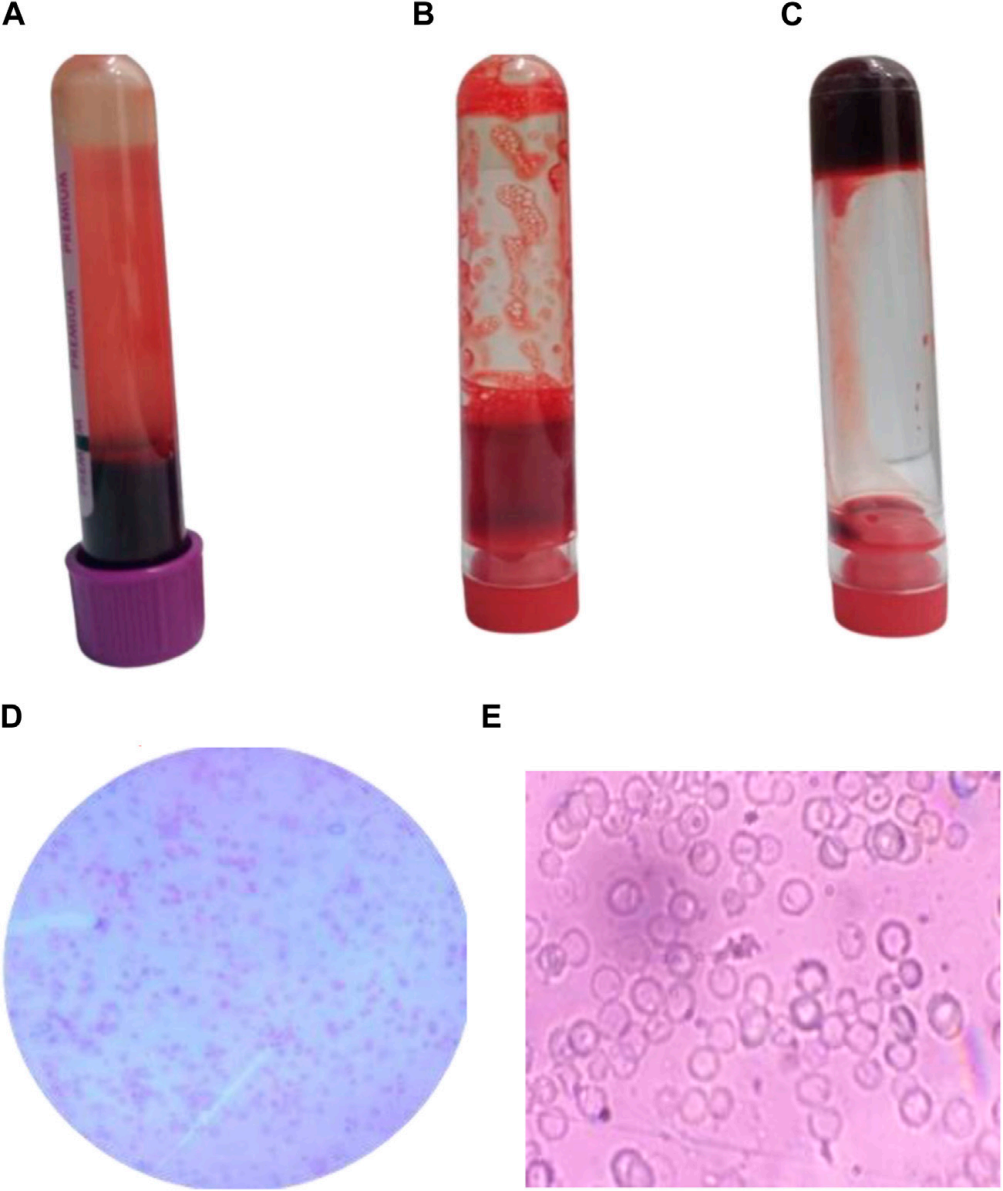
Anticoagulant activity of Ag/Ag_2_O NPs synthesized using O. europaea: **(A)** Blood sample in EDTA tube, **(B)** Blood sample treated with 150 ppm Ag/Ag₂O NPs, **(C)** Blood control samples without any additives, **(D, E)** Microscopic examination of blood samples treated with 150 ppm Ag/Ag_2_O NPs.

The anticoagulant activity of Ag/Ag₂O NPs was assessed to explore their potential as alternatives to traditional anticoagulants like EDTA (Figure 6). The results indicate that Ag/Ag₂O NPs effectively prevent blood coagulation, similar to the positive control, EDTA. This suggests that Ag/Ag₂O NPs could be a viable candidate for anticoagulant applications. Microscopic analysis of blood samples treated with Ag/Ag₂O NPs showed that red blood cells (RBCs) largely retained their biconcave disc shape, which is crucial for oxygen transport. However, slight deformation in RBC morphology was observed, possibly due to changes in pH or dilution effects. The effectiveness of Ag/Ag₂O NPs as anticoagulants is supported by their small particle size and high surface area, which enhance interaction with blood components and may improve anticoagulant performance (Talank et al., 2022).

Understanding the mechanisms behind the therapeutic effects of Ag/Ag₂O NPs is essential for realizing their full potential, particularly concerning their ability to scavenge reactive oxygen species (ROS) and interact with coagulation pathways (Kotcherlakota et al., 2019). While ROS are critical for many cellular functions, their excessive presence can lead to oxidative stress, which is implicated in numerous diseases, including cancer, cardiovascular disorders, and neurodegenerative conditions (Barabadi et al., 2023; Doudi et al., 2024). Ag/Ag₂O NPs have shown significant antioxidant properties, efficiently scavenging ROS at the cellular level. This activity helps mitigate oxidative damage, positioning these NPs as therapeutic agents capable of strengthening cellular defenses against oxidative stress (Dhir et al., 2024). By neutralizing harmful ROS, these NPs help maintain cellular homeostasis, enhancing cell health and resilience under toxic conditions. Furthermore, their antioxidant capacity may have a direct impact on platelet function and coagulation pathways, offering valuable insights into their potential role in managing thrombotic disorders (Barabadi et al., 2023).

### 3.7 Ag NP sub-acute toxicity study

The sub-acute toxicity study of Ag/Ag₂O NPs revealed a dose-dependent relationship in terms of safety and therapeutic potential. At a low dose of 2.5 mg/kg, the NPs displayed a relatively safe profile, with only mild, transient effects such as temporary fasting behavior, normal activity levels, and yellow urine. These effects resolved within 24 h, aligning with previous findings that lower doses of NPs tend to cause minimal and reversible adverse effects. In contrast, the higher dose of 5 mg/kg resulted in severe adverse outcomes, including mortality in 1 out of 5 animals, significant behavioral changes, dark-red urine, and severe ataxia. These findings indicate acute toxicity, consistent with reports in the literature that high doses of NPs can lead to pronounced neurotoxic effects and organ damage. Overall, this study highlights the narrow therapeutic window for Ag/Ag₂O NPs and emphasizes the need for careful dose management to avoid adverse effects (see Supplementary Table S2).

### 3.8 Ag/Ag_2_O NPs effect on metribuzin-induced hepatotoxicity and nephrotoxicity

In the next step, the effect of Ag/Ag_2_O NPs on metribuzin-induced liver and kidney toxicity was assessed in adult Wistar albino rats. To achieve this, the rats were divided into four groups (n = 5/group): Group I served as the control and received normal water; Group II was exposed to metribuzin for 21 days through drinking water to induce liver and kidney toxicity; Group III received Ag/Ag_2_O NPs via intraperitoneal injection for 21 days to evaluate the effects of the NPs by IP dose 0.0625 mg/kg b.w and Group IV was first exposed to metribuzin followed by intraperitoneal injection of Ag/Ag_2_O NPs used dose 0.125 mg/kg b.w for 21 days to assess the potential therapeutic effects of the NPs in mitigating metribuzin.

#### 3.8.1 Assessment of hematological parameters

Hematological parameters are key indicators of an organism’s health, particularly under toxic exposure and therapeutic interventions. This section examines the effects of metribuzin and Ag/Ag₂O NPs on WBC, RBC, and PLT counts, highlighting their role in countering metribuzin-induced hematotoxicity.

**FIGURE 7:**
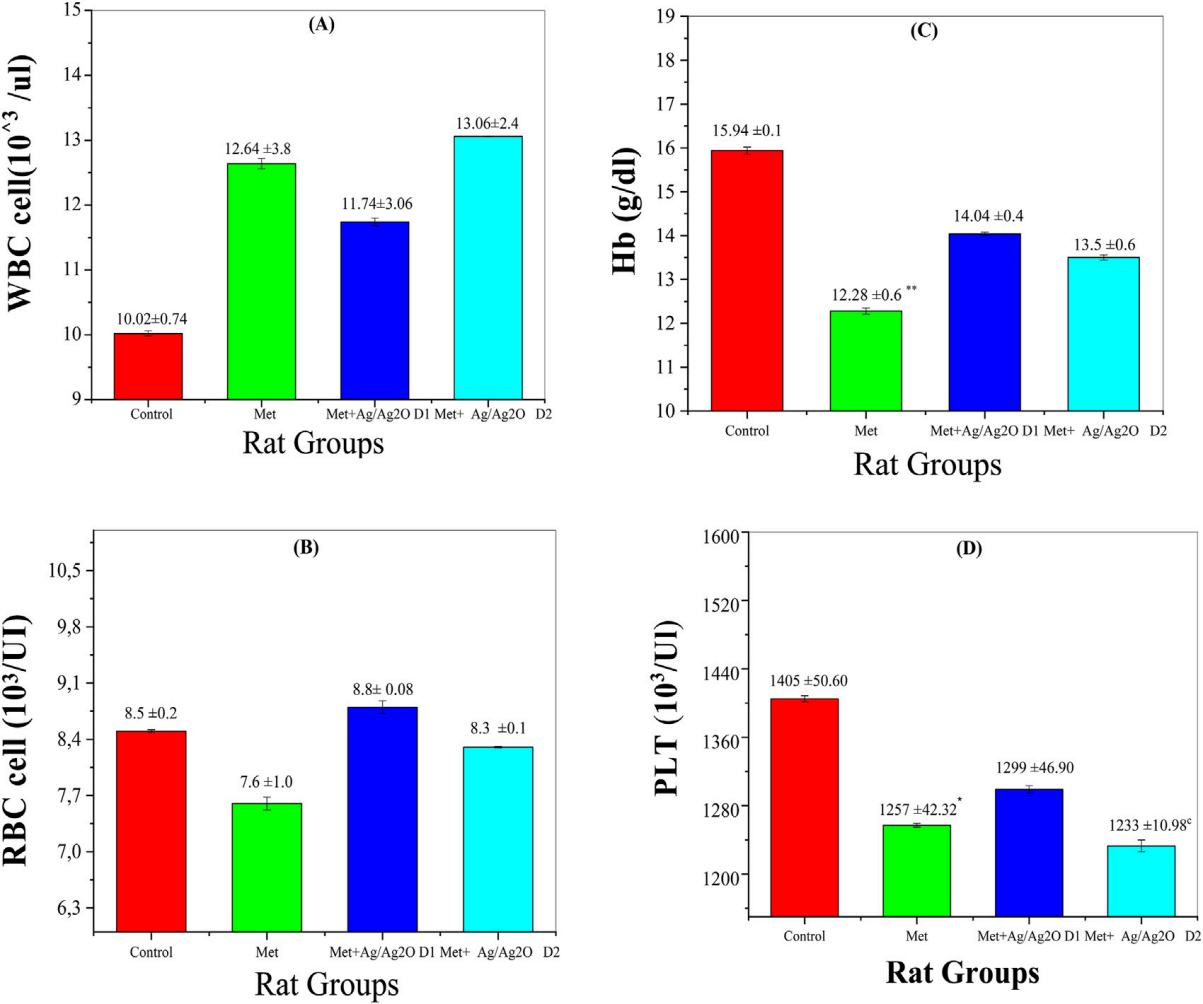
Comparison of serum of Haematological Parameters in the four rat groups. Group I (Control): Normal water consumption. Group II (Met): addition of Metribuzin (110 mg/kg body weight/day) in drinking water for 21 days. Group III (Met + Ag/Ag_2_O 0.062 mg/kg): Met exposure (as in Group II) followed by Ag/Ag_2_O NPs(dose 0.0625 mg/kg, body weight/day by intraperitoneal injection) for 21 days. Group IV (Met + Ag/Ag_2_O NPs0.125 mg/kg): Met exposure (as in Group II) followed by Ag/Ag_2_O NPs (dose 0.125 mg/kg, body weight/day by intraperitoneal injection) for 21 days. **(A)**: WBC cell 10^3^/ul, **(B)**: RBC cell 10^3^/UI, **(C)**: Hb (g/dL), **(D)**: PLT 10^3^/Ul. *p < 0.05, **p < 0.01, vs. Group I), ^c^ p < 0.001 vs. Group II.

Figure 7A shows the impact of metribuzin and Ag/Ag₂O NPs on White Blood Cell (WBC) counts. The control group (Group I) had normal WBC levels (10.02 ± 0.74 × 10³/µL). Metribuzin exposure (Group II) significantly raised WBC levels to 12.64 ± 3.8 × 10³/µL, indicating inflammation. The lower dose of Ag/Ag₂O NPs (0.062 mg/kg, Group III) reduced WBC counts to 11.74 ± 1.63 × 10³/µL, reflecting anti-inflammatory effects. However, the higher NP dose (0.125 mg/kg, Group IV) slightly increased WBC levels to 13.06 ± 2.46 × 10³/µL, suggesting that higher doses may still cause some inflammation or stress.

Figure 7B shows Red Blood Cell (RBC) counts across the experimental groups. The control group (Group I) had normal RBC levels (8.5 ± 0.21 × 10³/µL). Metribuzin exposure (Group II) significantly decreased RBC counts (7.62 ± 1.6 × 10³/µL), indicating anemia due to metribuzin’s hematotoxic effects. The lower dose of Ag/Ag₂O NPs (Group III) increased RBC levels to near-normal (8.8 ± 0.08 × 10³/µL), suggesting protective effects on red blood cell health. The higher NP dose (Group IV) improved RBC levels slightly (8.30 ± 0.12 × 10³/µL), but not as effectively as the lower dose, indicating possible toxicity at higher concentrations.

Figure 7D reveals the platelet (PLT) counts in the different groups. The control group (Group I) exhibited normal PLT counts (1,405 ± 50.6 × 10³/µL). Metribuzin exposure (Group II) led to a decrease in PLT levels (1,257 ± 42.32 × 10³/µL), indicative of thrombocytopenia, a common consequence of toxic exposure that affects bone marrow function. Administration of the lower dose of Ag/Ag₂O NPs (Group III) partially restored PLT counts to 1,299 ± 46.9 × 10³/µL, demonstrating a protective effect against metribuzin-induced thrombocytopenia. Conversely, the higher NP dose (Group IV) resulted in a significant drop in PLT counts (1,233 ± 10.98 × 10³/µL), suggesting potential platelet suppression or toxicity at higher nanoparticle concentrations. This outcome is supported by studies highlighting that while NPs offer therapeutic benefits, higher doses may impair platelet production or function.

To address this concern, comprehensive long-term studies are strongly recommended to assess the accumulation of Ag/Ag₂O NPs in various tissues and evaluate their biocompatibility over extended periods. These studies are crucial for fully understanding the potential effects of these Ag/Ag₂O NPs in therapeutic applications. By systematically tracking nanoparticle distribution and retention in vital organs, critical insights can be gained into possible adverse outcomes, such as chronic inflammation or oxidative stress. Furthermore, detailed investigations into long-term metabolic impacts, including changes in lipid metabolism and hematological parameters, will offer a more complete view of their safety profile (Roman et al., 2020). These future studies will significantly expand the understanding of the long-term implications of Ag/Ag₂O NPs, guiding the development of safer and more effective therapeutic applications. Rigorous assessments are essential for advancing nanoparticle-based treatments responsibly in clinical practice.

#### 3.8.2 Biochemical parameters

The biochemical parameters measured in this study provide insights into the metabolic impacts of metribuzin exposure and the potential ameliorative effects of Ag/Ag₂O NPs (NPs). The following figures illustrate these effects on key metabolic markers, including glucose, cholesterol, triglycerides, HDL, and LDL levels.

**FIGURE 8:**
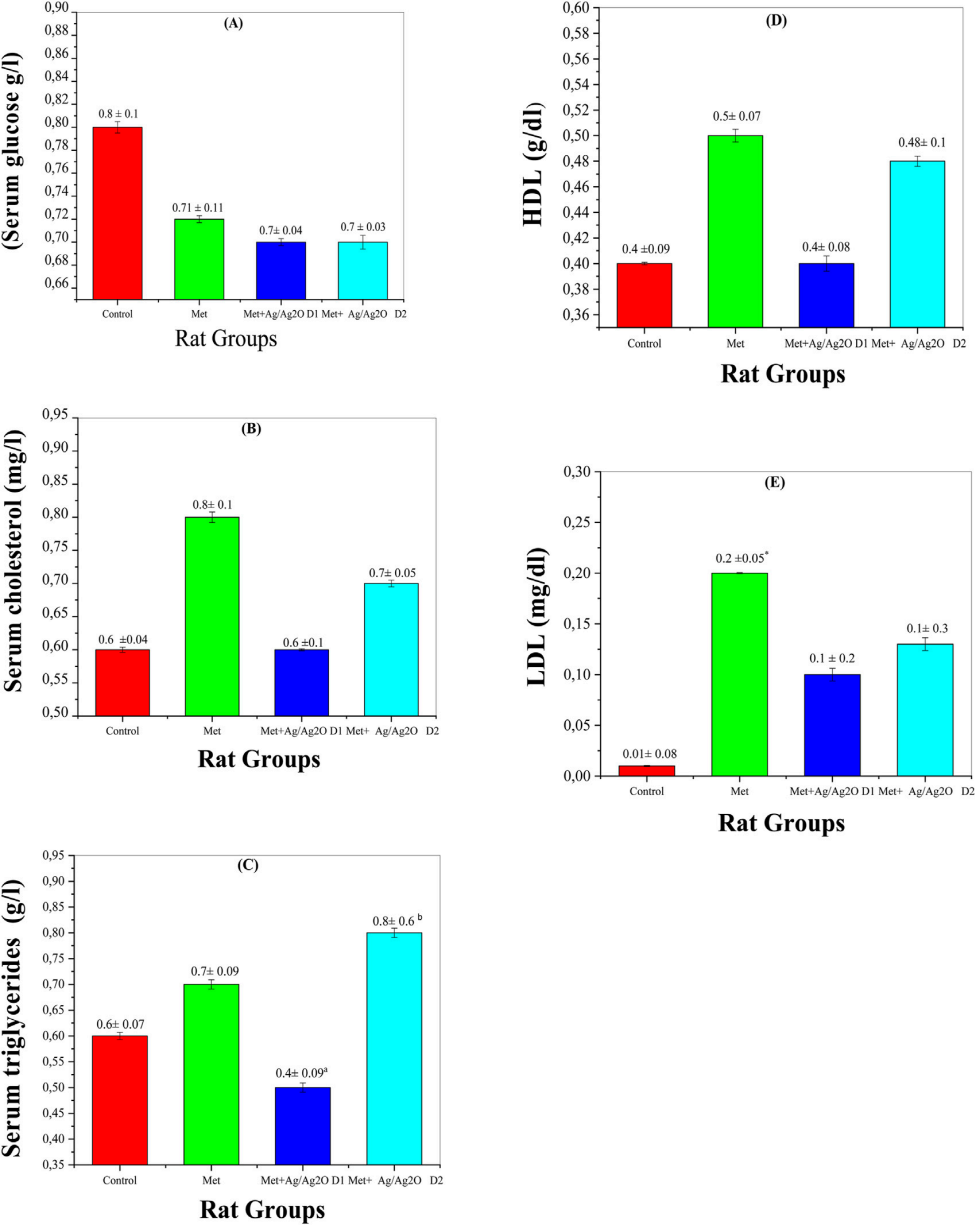
Comparison of serum biomarker levels in the four rat groups. Group I (Control): Normal water consumption. Group II (Met): addition of Metribuzin (110 mg/kg body weight/day) in drinking water for 21 days. Group III (Met + Ag/Ag_2_O NPs 0.062 mg/kg): Met exposure (as in Group II) followed by Ag/Ag_2_O NPs (dose 0.0625 mg/kg, body weight/day by intraperitoneal injection) for 21 days. Group IV (Met + Ag/Ag_2_O NPs0.125 mg/kg): Met exposure (as in Group II) followed by Ag/Ag2O NPs (dose 0.125 mg/kg, body weight/day by intraperitoneal injection) for 21 days. **(A)**: (Serum glucose g/L), **(B)**: Serum cholesterol (mg/L), **(C):** Serum triglycerides (g/L), **(D)**: HDL g/dL), **(E)**: LDL mg/dL. *p < 0.05, vs. Group I), a p < 0.05, b p < 0.01 vs. Group II.

Figure 8A demonstrates the effects of metribuzin on serum glucose levels. Metribuzin exposure resulted in a slight decrease in glucose levels (0.72 ± 0.12 g/L) compared to the control group (0.81 ± 0.1 g/L), suggesting a potential disruption in glucose homeostasis. This observation is consistent with research indicating that metribuzin can affect metabolic processes, potentially impacting glucose regulation (Yadav et al., 2024). Treatment with Ag/Ag₂O NPs (NPs) at a lower dose (0.062 mg/kg) slightly improved glucose levels to 0.74 ± 0.04 g/L, and the higher dose (0.125 mg/kg) also restored levels to 0.77 ± 0.03 g/L. This suggests that the antioxidant properties of the Ag/Ag₂O NPs may help stabilize glucose metabolism. The modest fluctuations in glucose levels across groups imply that metribuzin’s primary effects may be more pronounced in other metabolic areas, such as lipid metabolism (Medjdoub et al., 2011).

Figure 8B illustrates the effects of metribuzin and Ag/Ag₂O NPs on serum cholesterol levels. Metribuzin exposure led to an increase in serum cholesterol (0.83 ± 0.18 mg/L) compared to the control group (0.69 ± 0.04 mg/L), reflecting disrupted lipid metabolism and increased cardiovascular risk (Velisek et al., 2009). Treatment with a lower dose of Ag/Ag₂O NPs (0.062 mg/kg) reduced cholesterol levels to 0.64 ± 0.14 mg/L, indicating a protective effect against lipid dysregulation. At the higher NP dose (0.125 mg/kg), cholesterol levels remained somewhat elevated (0.77 ± 0.05 mg/L) compared to the low-dose group but were still lower than the metribuzin-only group. These results suggest that Ag/Ag₂O NPs, particularly at lower doses, may help normalize cholesterol levels and counteract metribuzin-induced dyslipidemia (Laib et al., 2024b).

Figure 8C shows serum triglyceride levels, which increased in the metribuzin group (0.68 ± 0.09 g/L) compared to the control (0.59 ± 0.07 g/L), indicating altered fat metabolism potentially due to hepatic dysfunction (Husak et al., 2016). Administration of Ag/Ag₂O NPs at the lower dose (0.062 mg/kg) significantly reduced triglyceride levels to 0.47 ± 0.09 g/L, suggesting an improvement in lipid metabolism. However, the higher NP dose (0.125 mg/kg) led to an unexpected rise in triglycerides (0.80 ± 0.61 g/L), implying that while lower NP doses are beneficial, higher doses may induce metabolic stress or adverse effects (Laib et al., 2024a). This underscores the importance of precise dose management to avoid negative impacts on fat metabolism. Figures 8D, 7E show that HDL levels remained stable across all experimental groups, indicating no significant impact on ‘good’ cholesterol from either metribuzin or Ag/Ag₂O NP treatments. HDL levels were consistent in the control (0.48 ± 0.09 g/L), metribuzin (0.50 ± 0.07 g/L), and both low- and high-dose Ag/Ag₂O NP groups. In contrast, LDL levels were significantly higher in the metribuzin group (0.194 ± 0.05 mg/L) compared to the control (0.092 ± 0.08 mg/L), indicating increased cardiovascular risk. Treatment with Ag/Ag₂O NPs reduced LDL levels, with lower doses being more effective (0.136 ± 0.26 mg/L for low dose; 0.13 ± 0.3 mg/L for high dose). This suggests that Ag/Ag₂O NPs can lower LDL levels and mitigate metribuzin-induced dyslipidemia, with lower doses showing more consistent lipid profile improvements.

The observed toxicity at higher doses of Ag/Ag₂O NPs illustrates a well-known issue where therapeutic benefits can transform into harmful effects as the dose increases. These NPs exhibit significant biological benefits at lower doses, such as scavenging free radicals and enhancing cellular defenses. However, when administered at higher doses, they may overwhelm the body’s natural regulatory systems, leading to oxidative stress, disturbances in lipid metabolism, and hematopoietic problems, such as dyslipidemia and thrombocytopenia (Reddy et al., 2012).

This highlights the narrow therapeutic window of Ag/Ag₂O NPs, making precise dose optimization essential. Due to their unique size and surface properties, NPs interact with biological systems in a way that can promote pro-oxidant activity at higher concentrations, causing cellular damage and platelet dysfunction.

Supporting studies, such as those by Iavicoli et *al*. (Iavicoli et al., 2018) and Reddy et al. (Reddy et al., 2012), demonstrate similar biphasic responses, where lower doses of NPs enhance healing or improve biological functions, while higher doses lead to toxicity. These findings reinforce the importance of carefully balancing the dosage to ensure the safety and efficacy of Ag/Ag₂O NPs.

Thus, while our results reveal the promising therapeutic potential of Ag/Ag₂O NPs at lower doses, the safety risks associated with higher concentrations necessitate further dose-response research. Future investigations should focus on approaches like nanoparticle surface modification or targeted delivery systems to expand the therapeutic window, ensuring improved safety and efficacy.

#### 3.8.3 Liver and kidney function biomarkers

Assessing liver and kidney function is crucial in understanding the systemic effects of metribuzin and the potential therapeutic benefits of Ag/Ag₂O NPs. The following figures illustrate the impact of metribuzin exposure and NP treatment on key biomarkers related to renal and hepatic function, providing insights into possible organ-specific effects and therapeutic outcomes.

**FIGURE 9:**
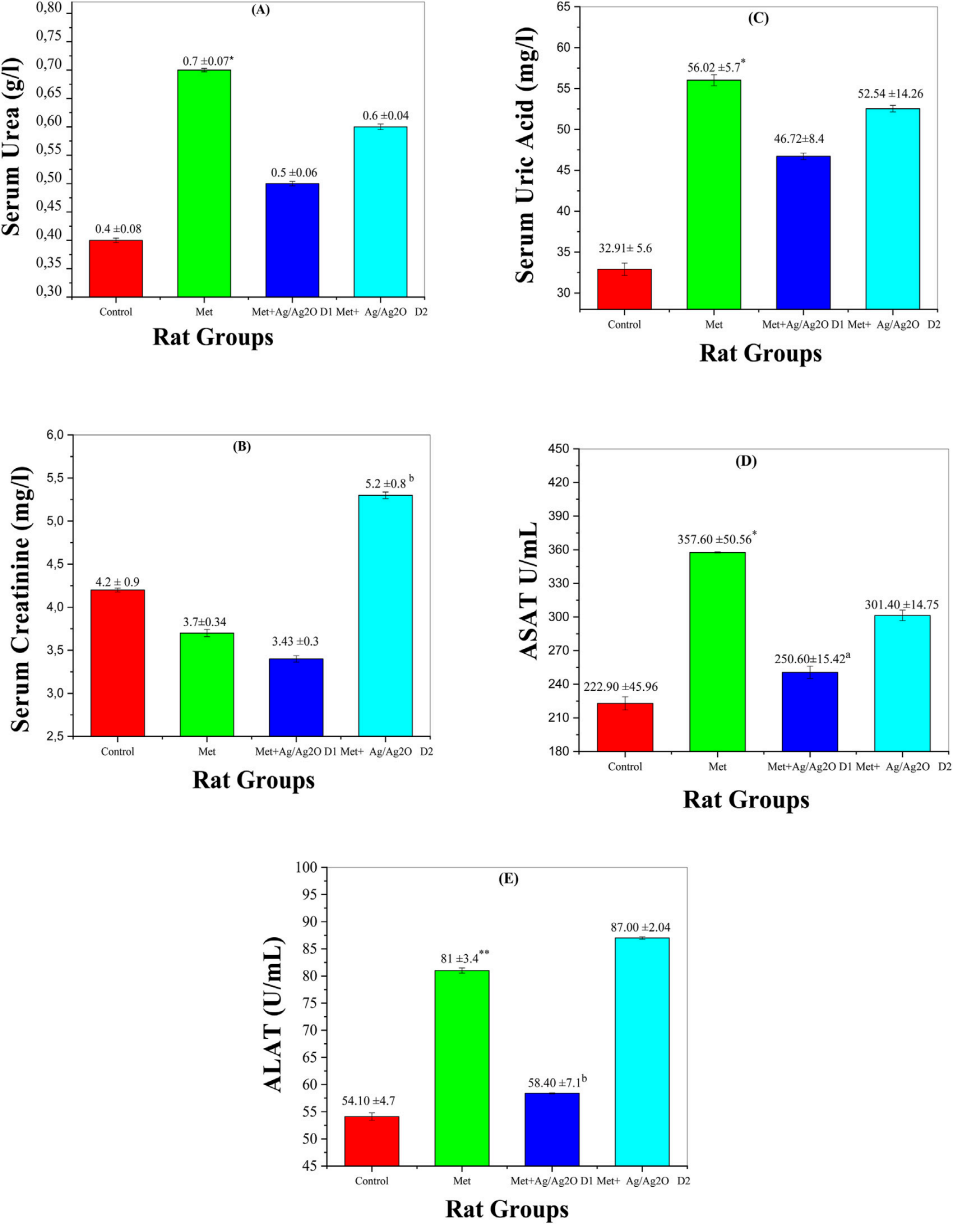
Comparison of liver and Kidney function biomarker levels in the four rat groups. Group I (Control): Normal water consumption. Group II (Met): addition of Metribuzin (110 mg/kg body weight/day) in drinking water for 21 days. Group III (8/Ag_2_O NPs0.062 mg/kg): Met exposure (as in Group II) followed by Ag/Ag_2_O NPs (dose 0.0625 mg/kg, body weight/day by intraperitoneal injection) for 21 days. Group IV (Met + Ag/Ag_2_O NPs0.125 mg/kg): Met exposure (as in Group II) followed by Ag/Ag_2_O NPs (dose 0.125 mg/kg, body weight/day by intraperitoneal injection) for 21 days. **(A)**: (Serum Urea g/L), **(B)**: Serum Creatinine mg/L, **(C)**: Serum Uric Acid mg/L **(D)**: ASAT U/mL, **(E)**: ALAT U/mL. *p < 0.05, **p < 0.01: significantly different from Group I, a p < 0.05, b p < 0.01: significantly different from Group II.

Figure 9A shows the impact of metribuzin on serum urea levels, a key marker of kidney function. Metribuzin exposure significantly elevated serum urea (0.67 ± 0.07 mg/dL) compared to the control group (0.43 ± 0.08 mg/dL), indicating impaired renal function, which is consistent with findings that metribuzin can induce nephrotoxicity and renal stress (Husak et al., 2016). Treatment with Ag/Ag_2_O NPs at a lower dose (0.062 mg/kg) reduced serum urea to 0.55 ± 0.06 mg/dL, suggesting a nephroprotective effect of the NPs. This aligns with literature suggesting that the Ag/Ag₂O NPs can mitigate renal damage by counteracting oxidative stress (Azzi et al., 2023). However, the higher NP dose (0.125 mg/kg) also reduced urea levels but the effect was less pronounced. This observation underscores the necessity of dose optimization for the Ag/Ag₂O NPs to achieve maximal renal protection, as higher doses may not proportionately enhance therapeutic effects and could potentially lead to reduced efficacy (Docea et al., 2020).

Figure 9B depicts serum creatinine levels, another important indicator of kidney function. Metribuzin exposure decreased creatinine levels (3.73 ± 0.34 mg/L) compared to the control (4.26 ± 0.9 mg/L). This reduction might reflect disrupted renal filtration capacity, as increased creatinine levels typically indicate impaired kidney function (Medjdoub et al., 2011). The lower dose of Ag/Ag2O NPs (0.062 mg/kg) improved creatinine levels slightly, suggesting partial restoration of kidney function. However, the higher NP dose (0.125 mg/kg) resulted in a significant increase in creatinine levels (5.28 ± 0.81 mg/L), exceeding even the control levels, which may indicate that excessive NP concentrations could induce additional renal stress or toxicity (Laib et al., 2023). This highlights the potential for high NP doses to exert adverse effects, necessitating careful dose management.

Figure 9C illustrates serum uric acid levels, which were significantly elevated in the metribuzin group (56.02 ± 5.72 mg/dL) compared to the control (32.91 ± 5.6 mg/dL), reflecting impaired renal function and altered purine metabolism, as elevated uric acid levels are commonly associated with kidney dysfunction (Chaib et al., 2023). The lower dose of Ag/Ag2O NPs (0.062 mg/kg) reduced uric acid levels to 46.72 ± 8.45 mg/dL, demonstrating a protective effect against metribuzin-induced nephrotoxicity. However, the higher NP dose (0.125 mg/kg) led to slightly elevated uric acid levels (52.54 ± 14.26 mg/dL) compared to the lower NP dose, indicating potential metabolic stress. This suggests that while Ag/Ag_2_O NPs offer some degree of protection, their effectiveness might be compromised at higher doses (Laib et al., 2024b).

Figure 9D shows ASAT (Aspartate Aminotransferase) levels, a marker of liver damage. Metribuzin exposure resulted in a significant increase in ASAT (357.6 ± 50.56 U/mL) compared to the control (222.9 ± 45.96 U/mL), indicating liver damage, as elevated ASAT levels are often associated with hepatotoxicity (Tayeb et al., 2012). The lower dose of Ag/Ag2O NPs (0.062 mg/kg) reduced ASAT levels to 250.6 ± 15.42 U/mL, reflecting a hepatoprotective effect. At the higher NP dose (0.125 mg/kg), ASAT levels were still elevated (301.4 ± 14.75 U/mL) but improved compared to the metribuzin-only group. This suggests that while Ag/Ag2O NPs can alleviate liver damage, higher doses may still leave some residual stress, emphasizing the need for dose optimization to enhance liver protection (Docea et al., 2020).

Figure 9E presents ALAT (Alanine Aminotransferase) levels, which were elevated in the metribuzin group (81 ± 3.47 U/mL) compared to the control group (54.1 ± 4.75 U/mL), indicating significant liver damage, as ALAT is a key marker for hepatocellular injury (Abdelmagid et al., 2023). The lower dose of Ag/Ag_2_O NPs (0.062 mg/kg) reduced ALAT levels to 58.4 ± 7.1 U/mL, demonstrating some liver protection. However, the higher NP dose (0.125 mg/kg) did not significantly decrease ALAT levels and even showed levels (87.0 ± 2.04 U/mL) comparable to the metribuzin group. This highlights that higher doses of NPs may not provide additional benefits and could potentially exacerbate liver damage, underlining the complexity of nanoparticle therapy and the critical need for optimizing NP dosage to avoid adverse effects (Anwar et al., 2021).

#### 3.8.4 Oxidative stress markers in liver and kidney

Oxidative stress markers provide valuable insights into the cellular damage and antioxidant status in response to metribuzin exposure and Ag/Ag₂O NP treatment. The following figures illustrate the impact of these substances on key biomarkers of oxidative stress, including malondialdehyde (MDA) and reduced glutathione (GSH), in both liver and kidney tissues. These biomarkers help to assess the extent of oxidative damage and the potential protective effects of Ag/Ag₂O NPs.

**FIGURE 10:**
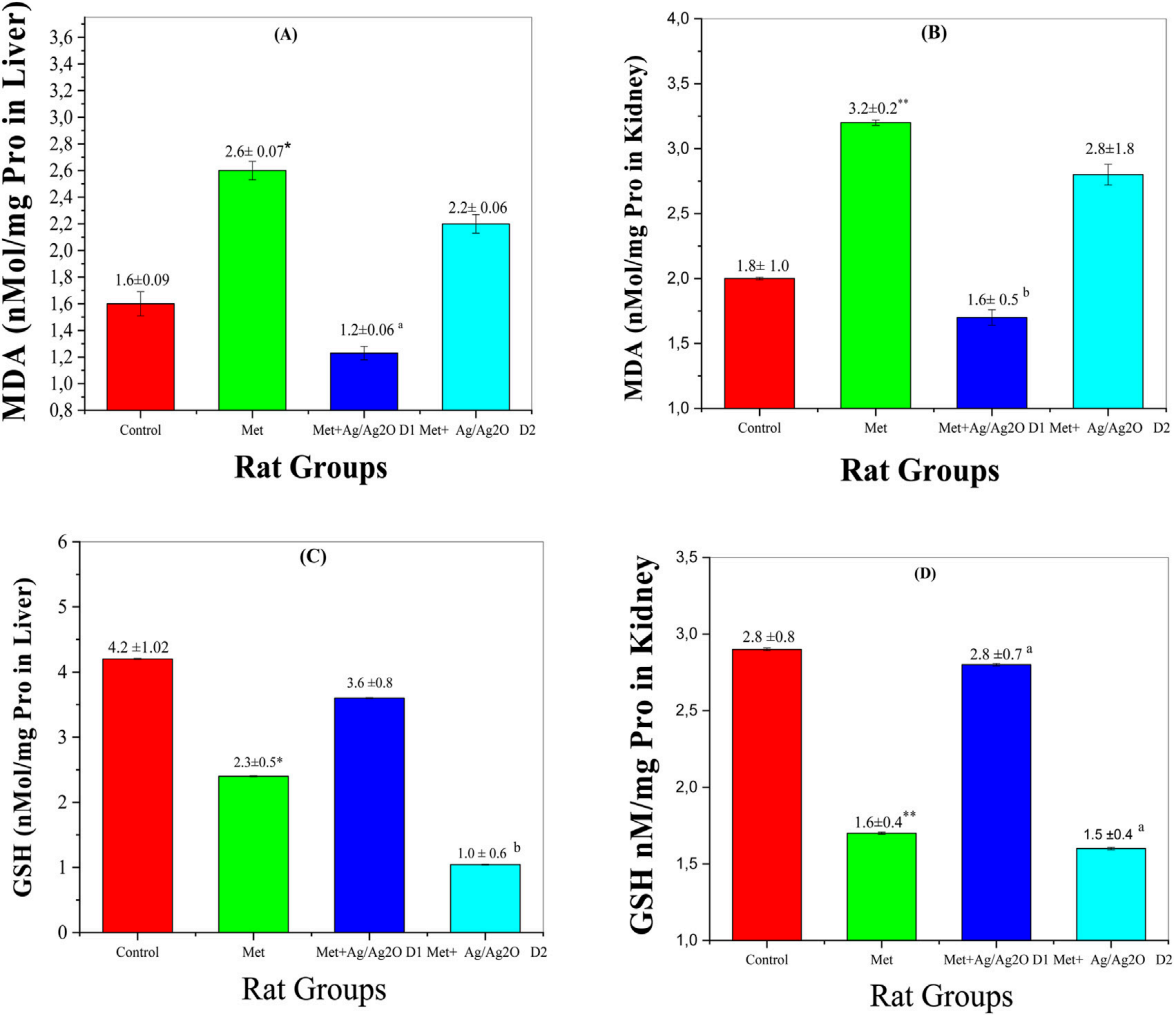
Comparison of oxidative stress biomarker levels in each of the liver and kidney in the four rat groups. Group I (Control): Normal water consumption. Group II (Met): addition of Metribuzin (110 mg/kg body weight/day) in drinking water for 21 days. Group III (Met + Ag/Ag_2_O NPs0.062 mg/kg): Met exposure (as in Group II) followed by Ag/Ag_2_O NPs (dose 0.0625 mg/kg, body weight/day by intraperitoneal injection) for 21 days. Group IV (Met + Ag/Ag_2_O NPs 0.125 mg/kg): Met exposure (as in Group II) followed by Ag/Ag_2_O NPs (dose 0.125 mg/kg, body weight/day by intraperitoneal injection) for 21 days. **(A)**: MDA nM/mg Pro in Liver, **(B)**: (MDA nM/mg Pro in Kidney), **(C)**: GSH nM/mg Pro in Liver, **(D)**: GSH nM/mg Pro in Kidney. *p < 0.05, **p < 0.01: significantly different from Group I, a p < 0.05, b p < 0.01: significantly different from Group II.

Figure 10 A demonstrates the effects of metribuzin and Ag/Ag₂O NPs on malondialdehyde (MDA) levels in the liver. MDA is a marker of lipid peroxidation, indicative of oxidative stress and cellular damage. In the control group (Group I), MDA levels were normal at 1.637 ± 0.92 nM/mg P, reflecting healthy liver function. Exposure to metribuzin (Group II) significantly increased MDA levels to 2.606 ± 0.78 nM/mg P, highlighting enhanced oxidative stress in the liver due to metribuzin-induced lipid peroxidation, as supported by literature documenting its pro-oxidant properties in hepatic tissues. Treatment with a lower dose of Ag/Ag₂O NPs (Group III) effectively reduced MDA levels to 1.234 ± 0.6 nM/mg P, suggesting that the NPs possess strong antioxidant properties capable of reducing oxidative damage in the liver. However, the higher dose of Ag/Ag₂O NPs (Group IV) resulted in only a moderate reduction in MDA levels (2.2 ± 0.6 nM/mg P), indicating that higher nanoparticle doses may be less effective or could even exacerbate oxidative stress. This emphasizes the importance of dose optimization to achieve therapeutic efficacy without causing additional stress to liver cells.

Figure 10B illustrates the effects on kidney MDA levels. The control group (Group I) had normal MDA levels (1.895 ± 1.00 nM/mg P). Metribuzin exposure (Group II) significantly increased MDA levels to 3.290 ± 0.21 nM/mg P, indicating severe oxidative stress and kidney damage. The lower dose of Ag/Ag₂O NPs (Group III) reduced MDA levels to 1.690 ± 0.55 nM/mg P, suggesting effective antioxidant protection. However, the higher dose (Group IV) raised MDA levels to 2.820 ± 1.82 nM/mg P, indicating that while lower doses of Ag/Ag_2_O NPs are protective, higher doses may exacerbate oxidative stress.

Figure 10C illustrates changes in reduced glutathione (GSH) levels in the liver, a crucial antioxidant defense molecule. The control group (Group I) showed healthy GSH levels (4.248 ± 1.02 nM/mg P), indicative of balanced redox status. Metribuzin exposure (Group II) significantly reduced liver GSH levels to 2.366 ± 0.56 nM/mg P, signifying impaired antioxidant capacity and increased vulnerability to oxidative stress, consistent with its known depletion of GSH in oxidative stress-related conditions. Treatment with the lower dose of Ag/Ag₂O NPs (Group III) substantially increased GSH levels to 3.621 ± 0.82 nM/mg P, indicating a restoration of antioxidant defenses due to the protective effects of Ag/Ag2O NPs. In contrast, the higher dose (Group IV) resulted in a marked reduction in liver GSH levels (1.044 ± 0.69 nM/mg P), further supporting the idea that excessive dosing may disrupt cellular antioxidant balance and lead to diminished therapeutic efficacy.

Figure 10D depicts GSH levels in the kidney, following a pattern similar to that seen in the liver. The control group (Group I) had normal GSH levels (2.853 ± 0.89 nM/mg P), reflective of stable renal antioxidant activity. Metribuzin exposure (Group II) significantly lowered kidney GSH levels to 1.670 ± 0.40 nM/mg P, indicating oxidative stress and compromised antioxidant defenses in the kidney. The lower dose of Ag/Ag₂O NPs (Group III) improved GSH levels to 2.810 ± 0.75 nM/mg P, suggesting that the NPs effectively restore renal antioxidant capacity and protect against metribuzin-induced oxidative damage (Endirlik et al., 2022). However, the higher dose (Group IV) led to a lesser improvement in GSH levels (1.540 ± 0.42 nM/mg P), reinforcing the notion that while Ag/Ag₂O NPs have therapeutic potential, their benefits are dose-dependent, and higher doses may hinder rather than enhance antioxidant defenses.

These findings highlight that Ag/Ag₂O NPs exert significant protective effects against oxidative stress in the liver and kidney at lower doses, likely due to their ability to neutralize reactive oxygen species (ROS) and enhance antioxidant defenses, including GSH production. However, the therapeutic window is narrow, as higher doses may reduce efficacy or even exacerbate oxidative damage, emphasizing the need for careful dose optimization in clinical applications.

#### 3.8.5 Histological alterations in liver and kidney

**FIGURE 11:**
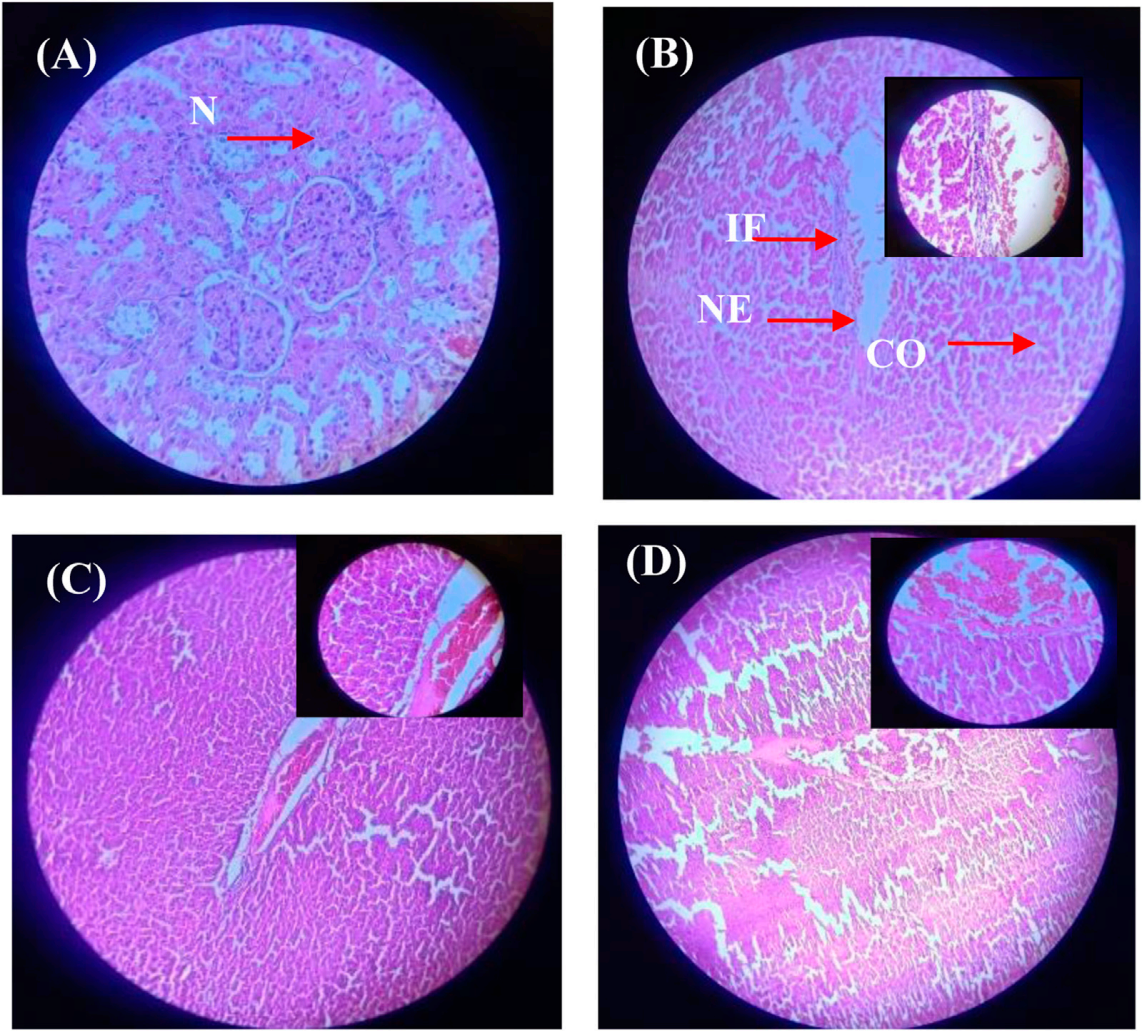
Micrographs of representative rat liver sections from different experimental groups showing the effect of metribuzin exposure and the protective effect of Ag/Ag2O NPs. **(A)** Liver section of a rat from Group I (control); **(B)** Liver section of a rat from Group II (metribuzin exposure); **(C)** Liver section from a rat of Group III (Ag/Ag_2_O NPs at 62.5 μg/kg after metribuzin exposure); **(D)** Liver section of a rat from Group IV (Ag/Ag_2_O NPs at 125 μg/kg after metribuzin exposure). N, normal hepatocyte; IF, Inflammatory cells; CO, Congestion of sinusoids; NE, Necrosis, × 40.

The histological evaluation of liver and kidney tissues across the experimental groups provides further insight into the protective effects of Ag/Ag₂O NPs against metribuzin-induced damage. In the liver (Figure 11; Supplementary Table S7), Group II, exposed to metribuzin, exhibited significant architectural damage, including extensive portal vein dilatation and congestion (+++), diffuse Kupffer cell proliferation (++), and moderate inflammatory cell infiltration (++). These findings suggest severe hepatic damage due to metribuzin’s toxic effects, consistent with its known hepatotoxicity linked to oxidative stress and inflammatory responses. However, rats treated with a lower dose of Ag/Ag₂O NPs (Group III) after metribuzin exposure showed considerable improvement, with only mild central vein dilation (+) and no evidence of necrosis or severe Kupffer cell proliferation. This indicates that the lower dose of Ag/Ag₂O NPs effectively mitigates hepatic damage, likely through its antioxidative and anti-inflammatory properties, as supported by literature highlighting the capacity of Ag/Ag2O NPs to stabilize cellular structures and reduce inflammation. On the other hand, the higher dose of Ag/Ag₂O NPs (Group IV) resulted in moderate central vein dilatation (++), coupled with persistent inflammatory cell infiltration (++), suggesting that while some protection was afforded, the higher dose may still induce additional stress or inflammation, highlighting the importance of dose optimization.

**FIGURE 12:**
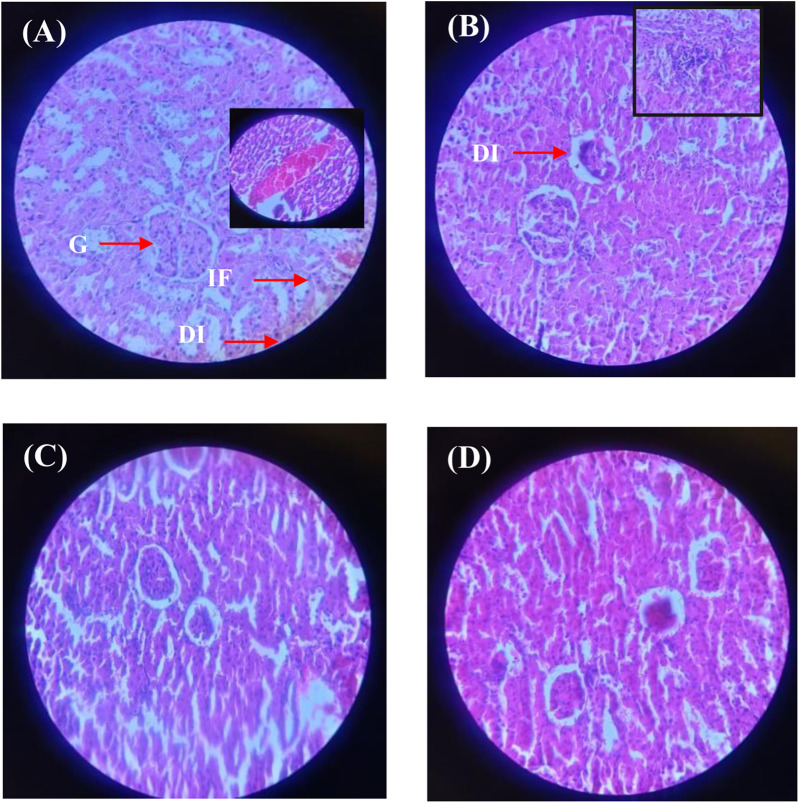
Micrographs of representative rat kidney sections from different experimental groups showing the effect of metribuzin exposure and the protective effect of Ag/Ag_2_O NPs. **(A)** Kidney section of a rat from Group I (control); **(B)** Kidney section of a rat from Group II (metribuzin exposure); **(C)** Kidney section from a rat of Group III (Ag/Ag_2_O NPs at 62.5 μg/kg after metribuzin exposure); **(D)** Kidney section of a rat from Group IV (Ag/Ag_2_O NPs at 125 μg/kg after metribuzin exposure). G, Glomeruli; T, Tubules; DI, Degenerative changes; A, Atrophy; C, Capsule distortion; IF, Inflammatory cells; × 40.

Similarly, the histopathological analysis of kidney tissues (Figure 12; Supplementary Table S8) demonstrated significant metribuzin-induced damage in Group II, with degenerative changes (+++), tubular dilation (++), and severe inflammatory cell infiltration (+++). These alterations reflect the nephrotoxicity of metribuzin, which is well-documented to induce oxidative stress and inflammation in renal tissues. Treatment with Ag/Ag₂O NPs at a dose of 62.5 μg/kg (Group III) after metribuzin exposure showed a marked reduction in these damage parameters, with better preservation of glomeruli and tubules, reduced tubular dilation (+), and minimal inflammatory infiltration (+), indicating a protective effect. The literature supports these findings, as Ag/Ag_2_O NPs have been shown to exert nephroprotective effects through their antioxidative and anti-inflammatory actions. However, the higher dose of Ag/Ag₂O NPs (Group IV) exhibited less protection, with moderate tubular dilation (++) and persistent inflammatory infiltration (++), suggesting that while some renal protection was observed, the higher dose may not be as effective as the lower dose in preventing tissue damage.

## 4 Conclusion

This study successfully demonstrated the protective effects of Ag/Ag₂O nanoparticles (NPs), synthesized using Olea europaea (olive) leaf extract, against metribuzin-induced toxicity in Wistar rats. Physicochemical characterization confirmed the successful formation of Ag/Ag₂O NPs loaded with phytochemicals, with crystallite sizes of 23 nm and 19 nm, particle size of 25 nm, and significant XRD, FTIR, and UV-Vis peaks indicative of Ag/Ag₂O formation. Metribuzin exposure significantly disrupted hematological and biochemical parameters, including elevated white blood cell (WBC) counts, reduced red blood cell (RBC) and hemoglobin (Hb) levels, and worsened lipid profiles (increased cholesterol, LDL, and triglycerides). Treatment with the lower NP dose (0.062 mg/kg) improved WBC, RBC, hemoglobin, and platelet counts, normalized lipid levels, and positively affected biochemical markers such as serum creatinine and uric acid. In contrast, the higher NP dose (0.125 mg/kg) showed mixed results, with some improvements but an increase in triglycerides and persistent elevations in ASAT and ALAT enzyme levels. These findings underscore the therapeutic potential of Ag/Ag₂O NPs in mitigating chemical toxicity, particularly in combating oxidative stress. Future research should focus on unraveling the mechanisms of protection offered by these NPs, assessing their long-term safety, and exploring their efficacy across diverse toxicity models. Further investigation into their clinical applications is also warranted to establish optimal dosing strategies that ensure both safety and therapeutic efficacy.

## 5 Declarations

Ethical Approval: Ethical approval statements for animal studies of this work were obtained for the study titled “Acute toxicity testing of Ag/Ag₂O NPs” from the University of El Oued, El Oued 39,000, Algeria, under the reference number 22/S.C/F.L.N.S/SE.U/2023, approved on 22/03/2023, with the study registered under reference number 14/2023.

Conflicts of Interest: The authors declare that there are no financial or personal conflicts of interest that could potentially influence or bias the outcomes of this research work.

## Data Availability

The original contributions presented in the study are included in the article/Supplementary Material, further inquiries can be directed to the corresponding author.
