# Supplementary material for: Antimutagenic and anticoagulant therapeutic effects of Ag/Ag2O nanoparticles from Olea europaea leaf extract: mitigating metribuzin-induced hepato-and nephrotoxicity
Source: Front Pharmacol. 2024 Oct 23;15:1485525. doi: 10.3389/fphar.2024.1485525 (PMC11538059; doi:10.3389/fphar.2024.1485525)
Supplement: Supplementary file 1 [file Table1.pdf]

Antimutagenic and Anticoagulant Therapeutic Effects of Ag/Ag_2O Nanoparticles from *Olea europaea* Leaf Extract: Mitigating Metribuzin-Induced Hepato- and Nephrotoxicity

Manel Azzi[1,2] ,Ibtissam Laib [2], Abderrhmane Bouafia [3,4] , Ifriqya Medila[1,2], Ali Tliba[5], Salah Eddine Laouini[3,4], Huda Alsaeedi[5], David Cornu[6], Mikhael Bechelany[6,7] and Ahmed Barhoum[8*]

[1]Laboratory of Biology, Environment and Health, Faculty of Natural and Life Sciences, University of El Oued, 39000, Algeria, manelaz@yahoo.fr, ifrqya-medila@univ-eloued.dz

[2] Department of Cellular and Molecular Biology, Faculty of Natural and Life Sciences, University of El Oued, 39000, El Oued, Algeria, laib-ibtissam@univ-eloued.dz.

[3]Department of Process Engineering and Petrochemical, Faculty of Technology, University of El Oued, El Oued 39000, Algeria, abdelrahmanebouafia@gmail.com, salah_laouini@yahoo.fr

[4] Laboratory of Biotechnology Biomaterials and Condensed Matter, Faculty of Technology, University of El Oued, El Oued 39000, Algeria,

[5] Lab. VTRS, Faculty of Technology, University of El Oued, El-Oued 3900, Algeria, TLIBA39@gmail.com

NanoStruc Research Group, Chemistry Department, Faculty of Science, Helwan University, Cairo, 11795, Egypt

[5] Department of Chemistry, College of Science, King Saud University, Riyadh, Saudi Arabia, halsaeedi@ksu.edu.sa

[6]Institut Européen des Membranes, IEM, UMR-5635, University Montpellier, ENSCM, CNRS, Place Eugene Bataillon, Montpellier, France, david.cornu@umontpellier.fr, mikhael.bechelany@umontpellier.fr

[7]Gulf University for Science and Technology, GUST, Helwan, Kuwait

[8]NanoStruc Research Group, Chemistry Department, Faculty of Science, Helwan University, Cairo, Egypt

Corresponding author: ahmed.barhoum@science.helwan.edu.eg

Supplementary information

Table S1. Antimutagenic activity of the Ag/Ag_2O NPs biosynthesized by aqueous extract of O. europaea leaves against 1-NP in S. typhimurium TA98.

Test Items	Concentration	Revertants TA98	Inhibition %
Positive control (1-NP)	200 ng/tube	1290 ± 9.8	/
Ag/Ag_2O NPs	50 µg/tube	987 ± 7.5	23.48%
	100 µg/tube	768 ± 3.6	40.46%
	150 µg/tube	679 ± 4.1	47.36%
	200 µg/tube	429 ± 9.8	66.74%
	250 µg/tube	374 ± 3.9	70%

Table S2: Mortality Rates, Behavioral Observations, and Clinical Manifestations Following Sub-Acute Toxicity Assessment of Ag/Ag_2O NPs

Ag/Ag_2O NPs	Time	Mortality	Feed/ water	Diarrhea	Color of urine	Lacrimation	Movements	Ataxie	Head twitches
Control	1h	0/5	Normal	-	Clear yellow	None	Normal	-	-
	2h		Normal	-	Clear yellow	None	Normal	-	-
	6h		Normal	-	Clear yellow	None	Normal	-	-
	12h		Normal	-	Clear yellow	None	Normal	-	-
	24h		Normal	-	Clear yellow	None	Normal	-	-
	2d		Normal	-	Clear yellow	None	Normal	-	-
	7d		Normal	-	Clear yellow	None	Normal	-	-
	14d		Normal	-	Clear yellow	None	Normal	-	-

	28d		Normal	-	Clear yellow	None	Normal	-	-
2.5 mg Ag/Ag_2O NPs/kg. bw	1h	0/5	Fasting	-	Clear yellow	None	Slow	-	-
	2h		Fasting	-	Dark yellow	None	Slow	-	-
	6h		Abnormal	+	Clear yellow	None	Abnormal	-	-
	12h		Abnormal	-	Clear yellow	None	Abnormal	-	-
	24h		Abnormal	-	Clear yellow	None	Normal	-	-
	2d		Normal	-	Clear yellow	None	Normal	-	-
	7d		Normal	-	Clear yellow	None	Normal	-	-
	14d		Normal	-	Clear yellow	None	Normal	-	-
	28d		Normal	-	Clear yellow	None	Normal	-	-
5 mg Ag/Ag_2O NPs/kg. bw	1h	1/5	Fasting	-	Clear yellow	None	Clumsy and invariably slow	+	+
	2h		Fasting	++	Dark-red	None	Clumsy and invariably slow	+++	++
	6h		Fasting	+	Dark yellow	None	Slow	++	++
	12h		Fasting	-	Yellow	None	Slow	+	++
	24h		Abnormal	-	Yellow	None	Abnormal	+	+
	2d		Abnormal	-	Yellow	None	Abnormal	+	-
	7d		Abnormal	-	Clear yellow	None	Normal	-	-
	14d		Normal	-	Clear yellow	None	Normal	-	-
	28d		Normal	-	Clear yellow	None	Normal	-	-

Table S3. Comparison of serum of Haematological Parameters in the four rat groups. Group I (Control): Normal water consumption. Group II (Met): addition of Metribuzin (110 mg/kg body weight/day) in drinking water for 21 days. Group III (Met+ Ag/Ag2O NPs0.062mg/Kg): Met exposure (as in Group II) followed by Ag/Ag2O NPs(dose 0.0625 mg/kg, body weight/day by intraperitoneal injection) for 21 days. Group IV (Met+ Ag/Ag2O NPs0.125 mg/Kg): Met exposure (as in Group II) followed by Ag/Ag2O NPs(dose 0.125 mg/kg, body weight/day by intraperitoneal injection) for 21 days. **(A)**: WBC cell10^3 /ul, **(B):** RBC cell 10^3/UI, **(C):** Hb (g/dl), **(D)**: PLT 10^3/Ul. *p < 0.05, **p < 0.01, vs Group I), ^{c} p < 0.001 vs Group II.

Rat groups	WBC cell(10^3 /ul)	RBC cell (10^6/UI)	Hb (g/dl)	PLT (10^3/Ul)
Group I (Control)	10.02± 0.74	8.5 ±0.21	15.94 ±0.17	1405 ±50.6
Group II Metribuzin	12.64 ±3.8	7.62 ±1.6	12.28 ±0.68**	1257 ±42.32*
Group III (MET+ Ag/Ag2O NPsdose 0.0625 mg/Kg b.w	11.74 ±1.63	8.8± 0.08	14.04 ±0.4	1299 ±46.9
Group IV MET+ Ag/Ag2O NPsdose 0.125 mg/Kg b.w	13.06 ±2.46	8.30 ±0.12	13.5 ±0.61	1233 ±10.98^{c}

Table S4. Comparison of serum biomarker levels in the four rat groups. Group I (Control): Normal water consumption. Group II (Met): addition of Metribuzin (110 mg/kg body weight/day) in drinking water for 21 days. Group III (Met+ Ag/Ag2O NPs0.062mg/Kg): Met exposure (as in Group II) followed by Ag/Ag2O NPs(dose 0.0625 mg/kg, body weight/day by intraperitoneal injection) for 21 days. Group IV (Met+ Ag/Ag2O NPs0.125 mg/Kg): Met exposure (as in Group II) followed by Ag/Ag2O NPs(dose 0.125 mg/kg, body weight/day by intraperitoneal injection) for 21 days. **(A):** (Serum glucose g/l), **(B)**: Serum cholesterol (mg/l), **(C):** Serum triglycerides (g/l), **(D)**: HDL g/dl), **(E):** LDL mg/dl. *p < 0.05, vs. Group I), a p < 0.05, b p < 0.01 vs Group II.

Rat groups	Serum glucose g/l	Serum Cholesterol mg/l	Serum Triglycerides g/l	HDL g/dl	LDL mg/dl
Group I (Control)	0.81± 0.1	0.69 ±0.04	0.59± 0.07	0.48 ±0.09	0.092± 0.08
Group II Metribuzin	0.72± 0.12	0.83± 0.18	0.68± 0.09	0.50± 0.07	0.194 ±0.05*
Group III (MET+ Ag/Ag2O NPsdose 0.0625 mg/Kg b.w	0.74± 0.04	0.64 ±0.14	0.47± 0.09[a]	0.41± 0.08	0.136± 0.26
Group IV MET+ Ag/Ag2O NPsdose 0.125 mg/Kg b.w	0.77± 0.03	0.77± 0.05	0.80± 0.61 [b]	0.48± 0.1	0.13± 0.3

Table S5.Comparison of liver and Kidney function biomarker levels in the four rat groups. Group I (Control): Normal water consumption. Group II (Met): addition of Metribuzin (110 mg/kg body weight/day) in drinking water for 21 days. Group III (Met+ Ag/Ag2O NPs0.062mg/Kg): Met exposure (as in Group II) followed by Ag/Ag2O NPs(dose 0.0625 mg/kg, body weight/day by intraperitoneal injection) for 21 days. Group IV (Met+ Ag/Ag2O NPs0.125 mg/Kg): Met exposure (as in Group II) followed by Ag/Ag2O NPs(dose 0.125 mg/kg, body weight/day by intraperitoneal injection) for 21 days. **(A):** (Serum Urea g/l), **(B):** Serum Creatinine mg/l, **(C):** Serum Uric Acid mg/l **(D):** ASAT U/mL, **(E):** ALAT U/mL. *p < 0.05, **p < 0.01: significantly different from Group I, a p < 0.05, b p < 0.01: significantly different from Group II.

Rat groups	Serum Urea g/l	Serum creatinine mg/l	Serum Uric Acid mg/l	ASAT U/mL	ALAT U/mL
Group I (Control)	0.43 ±0.08	4.26± 0.9	32.91± 5.6	222.9 ±45.96	54.1 ±4.75
Group II Metribuzin	0.67 ±0.07*	3.73 ±0.34	56.02 ±5.72*	357.6 ±50.56*	81 ±3.47**
Group III (MET+ Ag/Ag2O NPsdose 0.0625 mg/Kg b.w	0.55 ±0.06	3.43 ±0.38	46.72 ±8.45	250.6±15.42 [a]	58.4 ±7.1[b]
Group IV (MET+ Ag/Ag2O NPsdose 0.125 mg/Kg b.w)	0.59 ±0.04	5.28 ±0.81 [b]	52.54 ±14.26	301.4 ±14.75	87.0 ±2.04

Table S.6. Comparison of oxidative stress biomarker levels in each of the liver and kidney in the four rat groups. Group I (Control): Normal water consumption. Group II (Met): addition of Metribuzin (110 mg/kg body weight/day) in drinking water for 21 days. Group III (Met+

Ag/Ag2O NPs0.062mg/Kg): Met exposure (as in Group II) followed by Ag/Ag2O NPs(dose 0.0625 mg/kg, body weight/day by intraperitoneal injection) for 21 days. Group IV (Met+ Ag/Ag2O NPs0.125 mg/Kg): Met exposure (as in Group II) followed by Ag/Ag2O NPs(dose 0.125 mg/kg, body weight/day by intraperitoneal injection) for 21 days. **(A)**: MDA nM/mg Pro in Liver, **(B)**: (MDA nM/mg Pro in Kidney), **(C):** GSH nM/mg Pro in Liver, **(D):** GSH nM/mg Pro in Kidney. *$p < 0.05$, **$p < 0.01$: significantly different from Group I, a $p < 0.05$, b $p < 0.01$: significantly different from Group II.

Rat groups	**MDA nM/mg Pro in Liver**	**MDA nM/mg Pro in Kidney**	**GSH nM/mg Pro in liver**	**GSH nM/mg Pro in Kidney**
Group I (Control)	1.637±0.92	1.895± 1.00	4.248 ±1.02	2.853 ±0.89
Group II Metribuzin	2.606± 0.78 *	3.290±0.21**	2.366±0.56*	1.670±0.40**
Group III (MET+ Ag/Ag2O NPsdose 0.0625 mg/Kg b.w	1.234±0.6 [a]	1.690± 0.55 [b]	3.621 ±0.82	2.810 ±0.75 [a]
Group IV (MET+ Ag/Ag2O NPsdose 0.125 mg/Kg b.w)	2.214± 0.67	2.820 ±1.82	1.044± 0.69 [b]	1.540 ±0.42 [a]

Table S7. Micrographs of representative rat liver sections from different experimental groups showing the effect of metribuzin exposure and the protective effect of Ag/Ag2O NPs. (a) Liver section of a rat from Group I (control); (b) Liver section of a rat from Group II (metribuzin exposure); (c) Liver section from a rat of Group III (Ag/Ag2O NPs at 62.5 µg/kg after metribuzin exposure); (d) Liver section of a rat from Group IV (Ag/Ag2O NPs at 125 µg/kg after metribuzin exposure). N; normal hepatocyte, IF; Inflammatory cells, CO; Congestion of sinusoids, NE; Necrosis; × 40.

Rat groups	Group I	Group II	Group III	Group IV
Parameters	Control	Metribuzin	MET+ Ag/Ag2O NPsdose 0.0625 mg/Kg b.w	(MET+ Ag/Ag2O NPsdose 0.125 mg/Kg b.w)
Inflammatory infiltration	-	+++	-	+
Vein Dilatation And Congestion	-	+++	-	+
Tubular Dilation	-	++++	-	+

Necrosis		++++		+

Table S8. Micrographs of representative rat kidney sections from different experimental groups showing the effect of metribuzin exposure and the protective effect of Ag/Ag2O NPs. (a) Kidney section of a rat from Group I (control); (b) Kidney section of a rat from Group II (metribuzin exposure); (c) Kidney section from a rat of Group III (Ag/Ag2O NPs at 62.5 µg/kg after metribuzin exposure); (d) Kidney section of a rat from Group IV (Ag/Ag2O NPs at 125 µg/kg after metribuzin exposure). G; Glomeruli, T; Tubules, DI; Degenerative changes, A; Atrophy, C; Capsule distortion, IF; Inflammatory cells; × 40.

Rat groups	Group I	Group II	Group III	Group IV
Parameters	Control	Metribuzin	MET+ Ag/Ag2O NPsdose 0.0625 mg/Kg b.w	(MET+ Ag/Ag2O NPsdose 0.125 mg/Kg b.w)
Inflammatory infiltration	-	+++	-	+
Degenerative Changes	-	+++	-	+
Tubular Dilation	-	+++	-	+
